# Optimal exercise prescription for patients with coronary heart disease across cardiac rehabilitation phases: a systematic review and network meta-analysis

**DOI:** 10.3389/fspor.2026.1813426

**Published:** 2026-06-11

**Authors:** Chuyuan Qiao, Shenglei Yang, Yan Wang

**Affiliations:** School of Sports Medicine and Rehabilitation, Beijing Sport University, Beijing, China

**Keywords:** 6-min walk distance, cardiac rehabilitation, coronary heart disease, exercise training, network meta-analysis, peak oxygen uptake, rehabilitation phases

## Abstract

**Background:**

Exercise-based cardiac rehabilitation is delivered across distinct clinical phases, yet the optimal exercise modality for improving cardiorespiratory fitness and functional capacity may differ by phase. This systematic review and network meta-analysis compared the relative effects of common exercise modalities on peak oxygen uptake (VO_2_peak) and 6-min walk distance (6MWD) in patients with coronary heart disease across rehabilitation phases.

**Methods:**

English and Chinese databases were searched from inception to April 30, 2026. Randomized controlled trials enrolling adults with coronary heart disease and evaluating exercise interventions were eligible. Two reviewers independently screened studies, extracted data, and assessed risk of bias using the Cochrane risk-of-bias tool. A frequentist network meta-analysis synthesized direct and indirect evidence within each phase (phase I inpatient, phase II early outpatient, phase III long-term maintenance). Continuous outcomes were pooled as mean differences with 95% confidence intervals (VO_2_peak in mL·kg⁻¹·min⁻¹; 6MWD in meters). Global inconsistency testing guided the use of consistency vs. inconsistency models. Interventions were ranked using the surface under the cumulative ranking curve (SUCRA).

**Results:**

Eighty-seven trials (7,241 participants) were included, covering aerobic exercise (AE), resistance training (RT), combined aerobic plus resistance training (AE + RT), high-intensity interval training (HIIT), traditional Chinese exercise (TCE; e.g., Tai Chi/qigong/Baduanjin), aerobic exercise combined with traditional Chinese exercise (AE + TCE), and conventional therapy (CT). Evidence was phase-imbalanced (9 phase I, 39 phase II, and 40 phase III trials); one study contributed outcomes to both Phase II and Phase III analyses. In phase I, evidence was comparatively limited; for VO_2_peak TCE and AE showed the most favorable probability of benefit vs. CT. Phase II provided the most informative network for VO_2_peak with RT and HIIT ranking highest, followed by AE + TCE. In phase III, for 6MWD, AE + RT ranked highest, with AE and TCE also showing favorable ranking; for VO_2_peak maintenance-phase signals favored RT- and combined-training–based approaches.

**Conclusions:**

The relative effectiveness of exercise modalities appears phase-dependent. These findings support phase-tailored exercise prescription in coronary heart disease, while emphasizing the need for additional head-to-head trials—particularly in early inpatient rehabilitation—to strengthen comparative evidence.

**Systematic review registration:**

https://www.crd.york.ac.uk/prospero/display_record.php?ID=CRD420251156834, PROSPERO, identifier CRD420251156834.

## 1 Introduction

Coronary heart disease (CHD) remains one of the leading causes of death globally, particularly among the elderly population (1). The high incidence of cardiovascular events progressively compromises the cardiopulmonary function of CHD patients (2, 3). Cardiopulmonary or cardiorespiratory fitness (CRF) serves as a crucial indicator of cardiovascular and respiratory functional capacity, typically assessed using peak oxygen uptake (VO_2_peak) and the 6-min walk distance (6MWD) (4, 5). Research indicates that CRF is closely associated with all-cause mortality and cardiovascular event incidence, possessing independent prognostic value (6–8). Consequently, improving cardiopulmonary endurance in CHD patients has become a core objective in cardiac rehabilitation therapy (9).

Cardiac rehabilitation is a vital component of CHD management, aiming to enhance patients' physiological function, improve quality of life, and reduce recurrence risk through personalized interventions such as exercise training, nutritional guidance, and psychological support (4, 9, 10). Cardiac rehabilitation typically unfolds in three phases: Phase I (Inpatient) focuses on early mobilization and low-intensity, short-duration, segmented exercise during hospitalization; Phase II (Post-Discharge Outpatient) involves structured interventions centered on aerobic training, progressively incorporating resistance training to enhance cardiopulmonary endurance, muscle strength, and functional independence; Phase III is the long-term maintenance phase, emphasizing patient self-management (9, 11, 12). Based on individual circumstances and sustainability, patients independently engage in more diverse, sustained exercise, supplemented by follow-up and community support as needed to reinforce adherence (9, 12). Given significant differences in physiological status, cardiovascular function, and exercise tolerance among CHD patients across phases, optimal exercise intervention protocols should be tailored to each rehabilitation stage (11).

In addition to phase-specific exercise prescription, contemporary cardiac rehabilitation increasingly incorporates home-based, hybrid, and telerehabilitation models supported by digital technologies. Existing evidence suggests that home-based and centre-based cardiac rehabilitation can produce broadly comparable improvements in exercise capacity and health-related quality of life (13), while wearable sensor-assisted home-based programmes and structured telerehabilitation protocols may facilitate individualized monitoring, feedback, and safety management (14, 15). These delivery models do not change the exercise modality itself, but they may influence how AE, RT, HIIT, TCE, and combined programmes are implemented, supervised, and progressed across rehabilitation phases.

However, despite widespread recognition of cardiac rehabilitation's efficacy, considerable uncertainty persists regarding the selection of the “optimal exercise prescription” (4, 10). Existing research predominantly focuses on comparisons within specific phases or single intervention modalities, such as high-intensity interval training (HIIT), moderate-intensity continuous training (MICT), and resistance training (RT) (16–19). While these studies provide partial evidence for exercise prescription development, substantial gaps remain in comprehensive comparisons across different intervention modalities and rehabilitation phases (4, 9). Particularly, systematic multi-modal comparative analyses of the differential effects of various exercise patterns on cardiopulmonary endurance improvement are lacking, limiting the development of individualized exercise intervention plans in clinical practice (9, 10).

To address this gap, the present study systematically evaluated and compared the effects of different exercise modalities on cardiopulmonary endurance in patients with coronary heart disease using a network meta-analysis (NMA) (20, 21). Subgroup analyses were performed according to cardiac rehabilitation phase (Phase I, Phase II, Phase III) to examine whether the relative effects of exercise interventions differed across rehabilitation stages (9, 11). By integrating direct and indirect evidence across exercise modalities, this study aimed to provide a comparative evidence base for phase-tailored exercise prescription in cardiac rehabilitation (20, 21). The findings may help inform clinical decision-making regarding the selection of exercise modalities according to patients' rehabilitation phase (9, 11).

## 2 Materials and methods

### 2.1 Protocol and registration

This systematic review and network meta-analysis (NMA) was reported in accordance with the Preferred Reporting Items for Systematic Reviews and Meta-Analyses extension statement for network meta-analyses (PRISMA-NMA) guidelines (20). The study protocol was registered in the PROSPERO international systematic review registry (No: CRD420251156834).

### 2.2 Search strategy

Following the Preferred Reporting Items for Systematic Reviews and Meta-Analyses literature search extension (PRISMA-S), we systematically searched English and Chinese databases including PubMed, Embase, Web of Science, Cochrane Library, Scopus, China National Knowledge Infrastructure (CNKI), and Wanfang Database. The search period spanned from each database's inception to April 30, 2026. The search was restricted to studies published in English or Chinese, including peer-reviewed journal articles and publicly available dissertations indexed in Chinese academic databases. No unpublished or original individual participant data were used. The search strategy combined subject headings (e.g., MeSH, Emtree) with free-text terms to cover three core concepts: (1). Target population, including patients with coronary heart disease, myocardial infarction, PCI, CABG, etc.; (2). Interventions, focusing on structured exercise programs such as HIIT, MICT, RT, TCE, etc.; (3). Study type, limited to randomized controlled trials. Detailed search strategies and specific search terms for each database are provided in Supplementary Tables S1–S3.

### 2.3 Inclusion criteria

Eligibility criteria were developed according to the Population-Intervention-Comparator-Outcome-Study design (PICOS) framework. Eligible participants were adults aged ≥18 years with coronary heart disease (CHD), including myocardial infarction, angina pectoris, and patients undergoing percutaneous coronary intervention (PCI) or coronary artery bypass grafting (CABG). Participants were required to be in a clearly defined cardiac rehabilitation phase: Phase I referred to rehabilitation during hospitalization; Phase II referred to early post-discharge rehabilitation occurring 2 weeks to 3 months after onset or surgery; and Phase III referred to long-term maintenance rehabilitation occurring 3 months or more after onset or surgery. If the original study provided a clear phase definition, that definition was prioritized. Eligible interventions were structured exercise prescriptions with clear specifications for exercise type, duration, intensity, or frequency, including aerobic exercise (AE), resistance training (RT), combined aerobic and resistance training (AE + RT), high-intensity interval training (HIIT), traditional Chinese exercise (TCE; e.g., Tai Chi, Qigong, or Baduanjin), or combined aerobic exercise and traditional Chinese exercise (AE + TCE). Comparators included conventional therapy (CT), standard drug therapy, or health education without structured exercise. The primary outcomes were cardiorespiratory fitness and functional capacity, assessed using peak oxygen uptake (VO_2_peak) and/or 6-min walk distance (6MWD). VO_2_peak, commonly measured by cardiopulmonary exercise testing (CPET), is considered the gold-standard physiological indicator of cardiorespiratory fitness. The 6MWD was included not as a direct substitute for VO_2_peak but as a complementary and practical measure of functional capacity because it is widely used in cardiac rehabilitation and reflects walking-based activities relevant to daily life. Previous systematic reviews have reported that the 6-min walk test is responsive to clinical change after outpatient cardiac rehabilitation and shows acceptable validity and reliability compared with exercise testing or CPET-derived peak VO_2_ (22, 23). Therefore, using both VO_2_peak and 6MWD allowed us to capture both physiological cardiorespiratory fitness and clinically relevant functional capacity. Extracted post-intervention data were required to be available or convertible to mean ± standard deviation. Eligible studies were randomized controlled trials (RCTs) with at least 10 participants per group and an intervention duration of at least 4 weeks or no fewer than 12 structured training sessions.

### 2.4 Exclusion criteria

Exclusion criteria are as follows: (1) Animal studies, *in vitro* research; conference abstracts, book chapters, reviews/commentaries; unpublished studies. (2) Non-coronary heart disease (CHD) populations, or mixed populations where CHD-specific data cannot be extracted. (3) Interventions primarily focused on breathing exercises, or combined interventions involving exercise alongside dietary or other non-exercise components where exercise effects could not be isolated; non-structured, non-progressive activities such as physical therapy or stretching. (4) Studies with incomplete data or unable to extract valid outcome measures.

### 2.5 Exercise intervention node configuration

To ensure network transferability, exercise modalities were categorized according to the FITT principles (Supplementary Table S4). When a single study included multiple eligible intervention groups (e.g., MICT vs. HIIT vs. UC), all relevant groups were incorporated into the network. For aerobic exercises of varying intensities, those not meeting HIIT criteria (i.e., high-intensity interval training) were classified as AE. Where original reports were unclear, two researchers independently determined classification based on exercise prescription details.

### 2.6 Literature screening and data extraction

Two researchers (Qiao CY and Yang SL) independently screened studies based on inclusion and exclusion criteria. After removing duplicates using Zotero software, initial screening was performed by reviewing titles and abstracts to exclude significantly irrelevant studies. Full-text articles were then read, and a rigorous re-screening based on the PICOS framework was conducted to determine the final inclusion list. Disagreements during screening were resolved through discussion with a third researcher.

Key data were extracted using a pre-designed standardized form, including: (1) Study details: first author name and publication year; (2) Participant characteristics: country, sample size, age, gender, and rehabilitation phase; (3) Intervention details: intervention type, training frequency, duration, and exercise intensity; (4) Primary outcome measures.

### 2.7 Risk of bias in individual studies

Two researchers (Qiao CY and Yang SL) independently assessed study quality using the Cochrane risk of bias tool (Cochrane Handbook, version 5.1.0) (24). Considering the difficulty of blinding participants and practitioners in exercise interventions, only six other risk of bias categories were evaluated: (1) random sequence generation; (2) allocation concealment; (3) blinding of outcome assessors; (4) completeness of outcome data; (5) selective reporting of results; (6) other sources of bias. Each study's risk of bias was ultimately rated as “low risk,” “high risk,” or “unclear.”

Additionally, the primary outcome measures (VO_2_peak, 6MWD) underwent evidence quality grading using the GRADE network meta-analysis extension (25). The assessment encompassed: (1) study limitations; (2) inconsistency; (3) indirectness; (4) imprecision; (5) publication bias. By evaluating the quality of both direct and indirect evidence, the certainty of evidence was ultimately categorized into four levels—“high,” “moderate,” “low,” or “very low”—to objectively assess the reliability of the analysis results.

### 2.8 Data analysis

This study employed a network meta-analysis using a frequentist framework with Stata 16.0 software (21) and RStudio 4.6. Primary outcome measures (VO_2_peak and 6-min walk distance) were continuous variables, with results presented as mean differences and their 95% confidence intervals. All extracted data were converted to uniform units: VO_2_peak to mL·kg⁻¹·min⁻¹ and 6-min walk distance to meters. When original studies did not directly report means or standard deviations, estimates were calculated using the formula recommended in the *Cochrane* Handbook based on medians and interquartile ranges (IQR) (26, 27).

To ensure transferability and reduce heterogeneity, independent evidence networks were constructed for each phase of cardiac rehabilitation (Phase I, Phase II, Phase III). Network diagrams were generated to visualize direct and indirect comparative relationships among interventions. Global consistency testing assessed overall inconsistency in the network analysis (28). Given the largely star-shaped distribution of the evidence network without effective closed loops, local assessments such as node splitting were not performed. This evidence structure also indicated that most active exercise modalities were connected primarily through CT rather than through direct head-to-head comparisons, which should be considered when interpreting comparative rankings. If the global consistency test yielded *P* *≥* 0.05, the consistency model was used for primary analysis; if *P* < 0.05, the inconsistency model was applied. The relative efficacy of each exercise intervention was assessed using the area under the cumulative ranking probability curve (SUCRA), where higher values (closer to 100%) indicate a greater probability of favorable ranking rather than definitive clinical superiority (29). To validate the robustness of conclusions, sensitivity analysis was conducted by excluding studies with high risk of bias to observe shifts in SUCRA rankings. Corrected funnel plots assessed small-sample effects or publication bias (30). Statistical significance was set at *P* < 0.05. To enhance clinical interpretability, the minimum clinically important difference (MCID) for 6MWD was prespecified as 25 meters (31).

## 3 Results

### 3.1 Literature screening process and results

**Figure 1:**
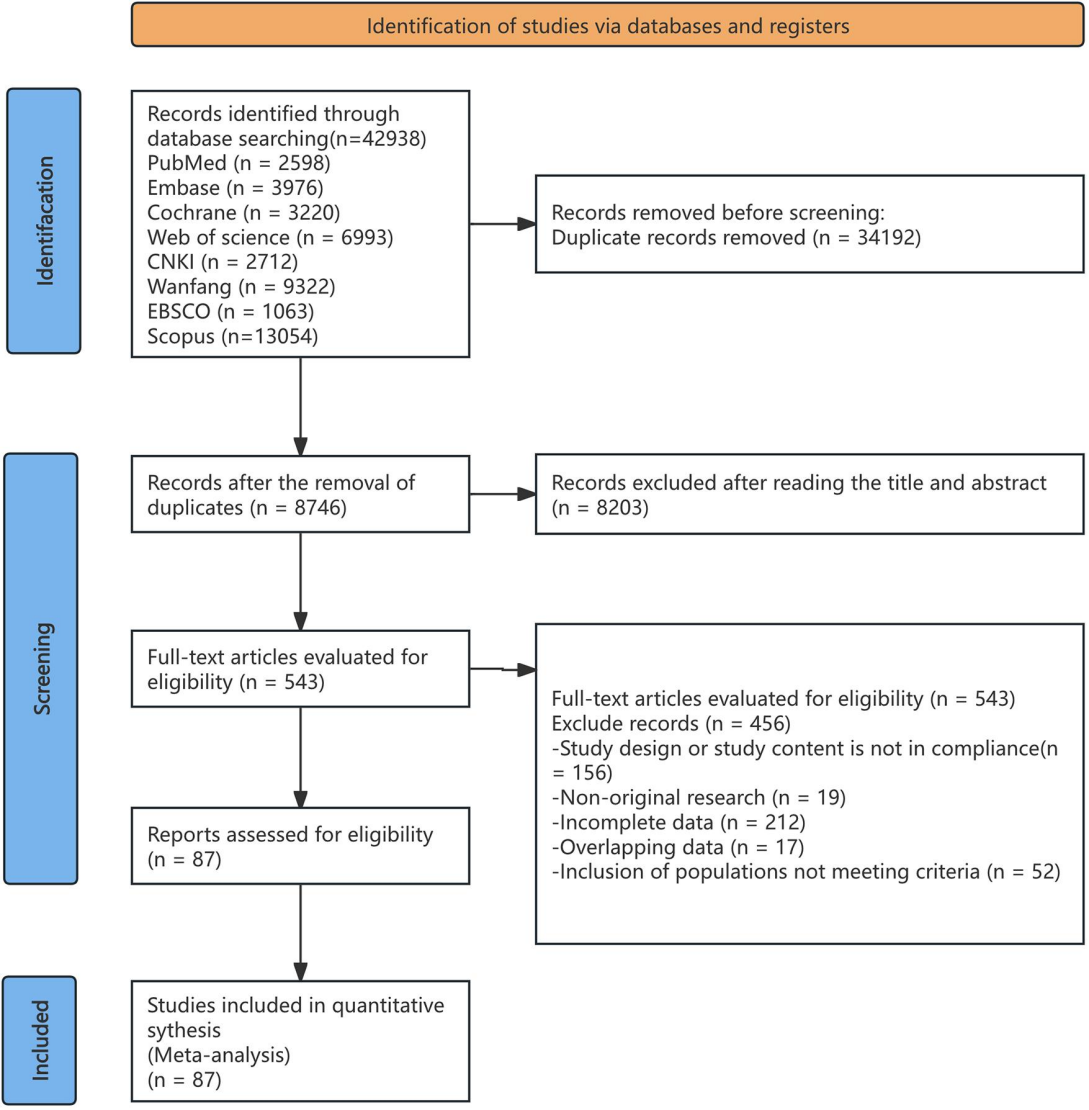
PRISMA 2020 flow diagram of study selection. A total of 87 randomized controlled trials (RCTs) were included in this network meta-analysis (NMA).

This study retrieved 42,938 records from nine databases. After removing duplicates, 8,746 records proceeded to the title and abstract screening phase. We excluded 8,203 records that did not meet the inclusion criteria. Subsequently, 543 full-text articles were evaluated, of which 456 were excluded: 156 due to non-compliant study design or content, 19 being non-original research, 212 lacking key information or outcome data, 17 with overlapping or duplicated data and 52 with non-compliant study populations. Ultimately, 87 randomized controlled trials met the requirements and were included in this network meta-analysis. The detailed search process is illustrated in Figure 1.

### 3.2 Characteristics of included studies

This study ultimately included 87 randomized controlled trials (RCTs) involving 7,241 patients with coronary heart disease. Among them, 3,649 patients were in the experimental group and 3,592 in the control group. The gender distribution was 4,503 males, 2,416 females, and 322 cases with unreported gender. These studies were published between 2001 and 2026, primarily originating from China (66 studies), other Asian countries (4 studies), and European, American, and Oceanian countries (17 studies). The mean age of participants was 61.52 ± 10.74 years. Based on cardiac rehabilitation phases, the included studies were distributed as follows: 9 studies in Phase I (inpatient) (32–40), 39 studies in Phase II (early outpatient) (41–79), and 40 studies in Phase III (maintenance) (12, 68, 80–117). Among them, 1 study reported the outcome indicators of both Phase II and Phase III simultaneously (68).

The studies encompassed 6 exercise intervention modalities. Aerobic exercise (AE) was the most prevalent intervention (*n* = 40), followed by AE combined with resistance training (AE + RT, *n* = 22), traditional Chinese exercises (TCE, *n* = 9), high-intensity interval training (HIIT, *n* = 7), AE combined with TCE (AE + TCE, *n* = 6), and resistance training alone (RT, *n* = 3).Regarding outcome measures, 40 studies reported VO_2_peak only, 34 reported 6MWD, and 13 reported both measures. Detailed baseline characteristics of all included trials are summarized in Supplementary Table S5.

### 3.3 Quality assessment of included studies

**Figure 2:**
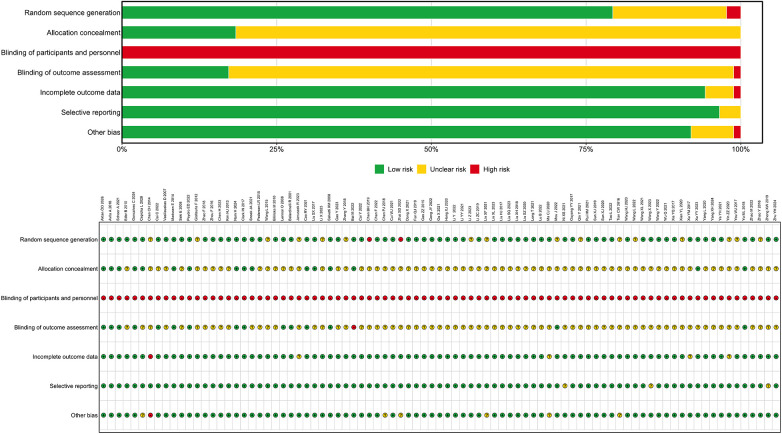
Risk of bias assessment.

Methodological quality of the 87 included studies was assessed using the *Cochrane* risk of bias tool. Regarding randomization processes, most studies (*n* = 69) were rated as low risk; 16 were rated as “unclear” due to unclear random sequence generation methods; and 2 were rated as high risk (studies did not specify randomization methods). Allocation concealment reporting was inadequate; only 16 studies confirmed reliable methods (e.g., centralized randomization or sealed envelopes), while 71 were rated “unclear” due to insufficient details. For blinding of participants, all studies were rated as high risk (*n* = 87), mainly because exercise-based interventions are difficult to blind in clinical practice. Regarding outcome blinding, 15 studies reported adequate blinding measures (e.g., assessment by independent evaluators) and were rated low risk; 71 studies were rated “unclear” due to lack of relevant information; one study was rated high risk for explicitly stating no blinding was used. Regarding missing outcome data, most studies (*n* = 82) performed well and were rated low risk. For selective reporting bias, 84 studies were rated low risk; other sources of bias (e.g., implementation and analysis bias) were also rated low risk in 80 studies. Overall, the included studies demonstrated acceptable quality in randomization and data completeness, though deficiencies in allocation concealment and blinding reporting may introduce uncertainty. These limitations were considered in the subsequent certainty of evidence (GRADE) assessment. Detailed risk of bias assessments are shown in Figure 2.

### 3.4 Network meta-analysis

**Figure 3:**
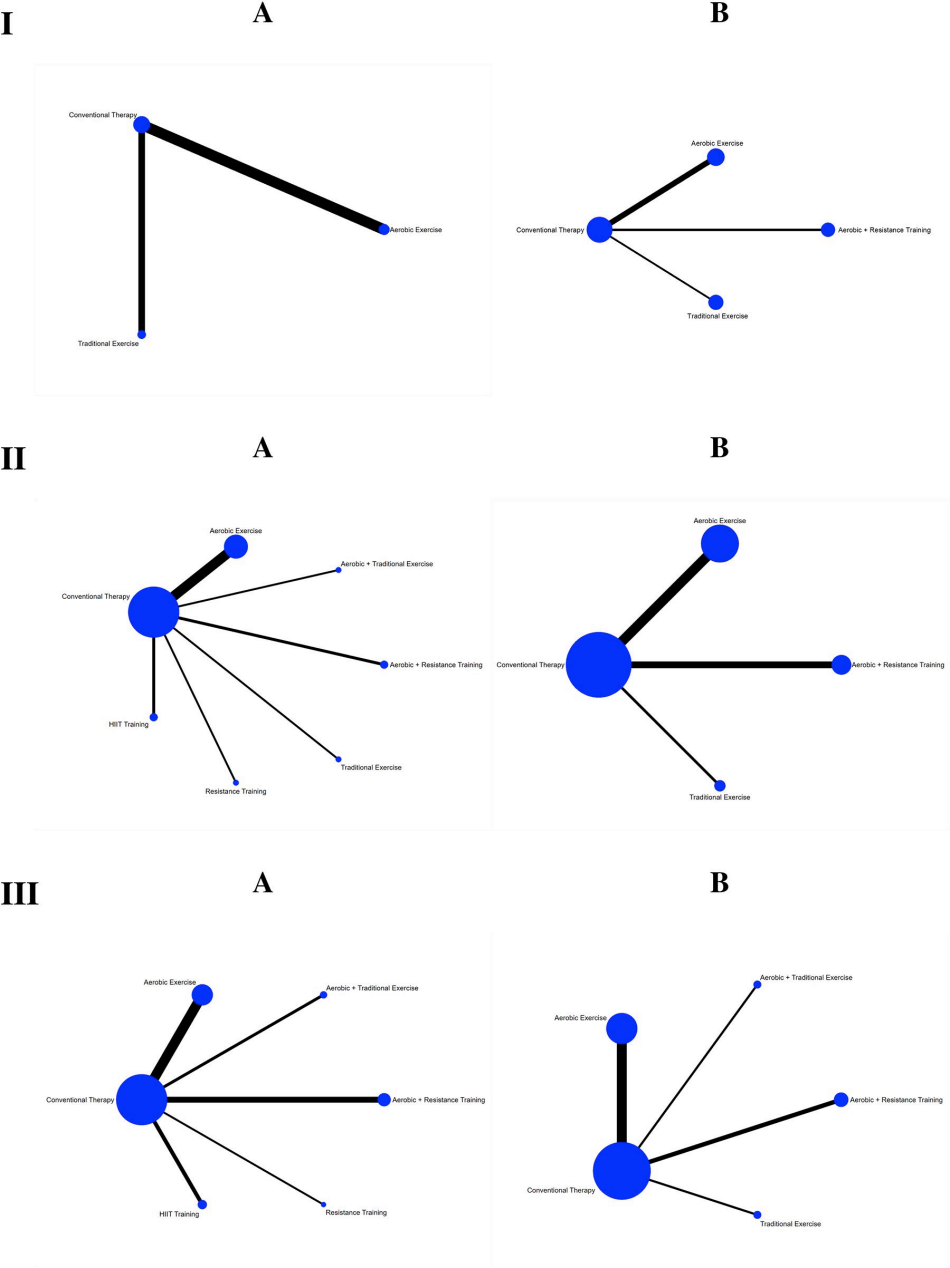
Network evidence plots by cardiac rehabilitation phase: **(A)** VO_2_peak, **(B)** 6MWD.

The complete network meta-analysis plot is shown in Figure 3.

#### 3.4.1 Effects of exercise on CRF in phase I CHD

This study assessed both direct and indirect evidence for Phase I indicators. Results showed a global inconsistency test *P* < 0.05 for both VO_2_peak and 6MWD, indicating significant inconsistency effects between studies. Therefore, an inconsistency model was used for meta-analysis. The details are shown in Supplementary Table S6.

##### 3.4.1.1 VO_2_peak

**Figure 4:**
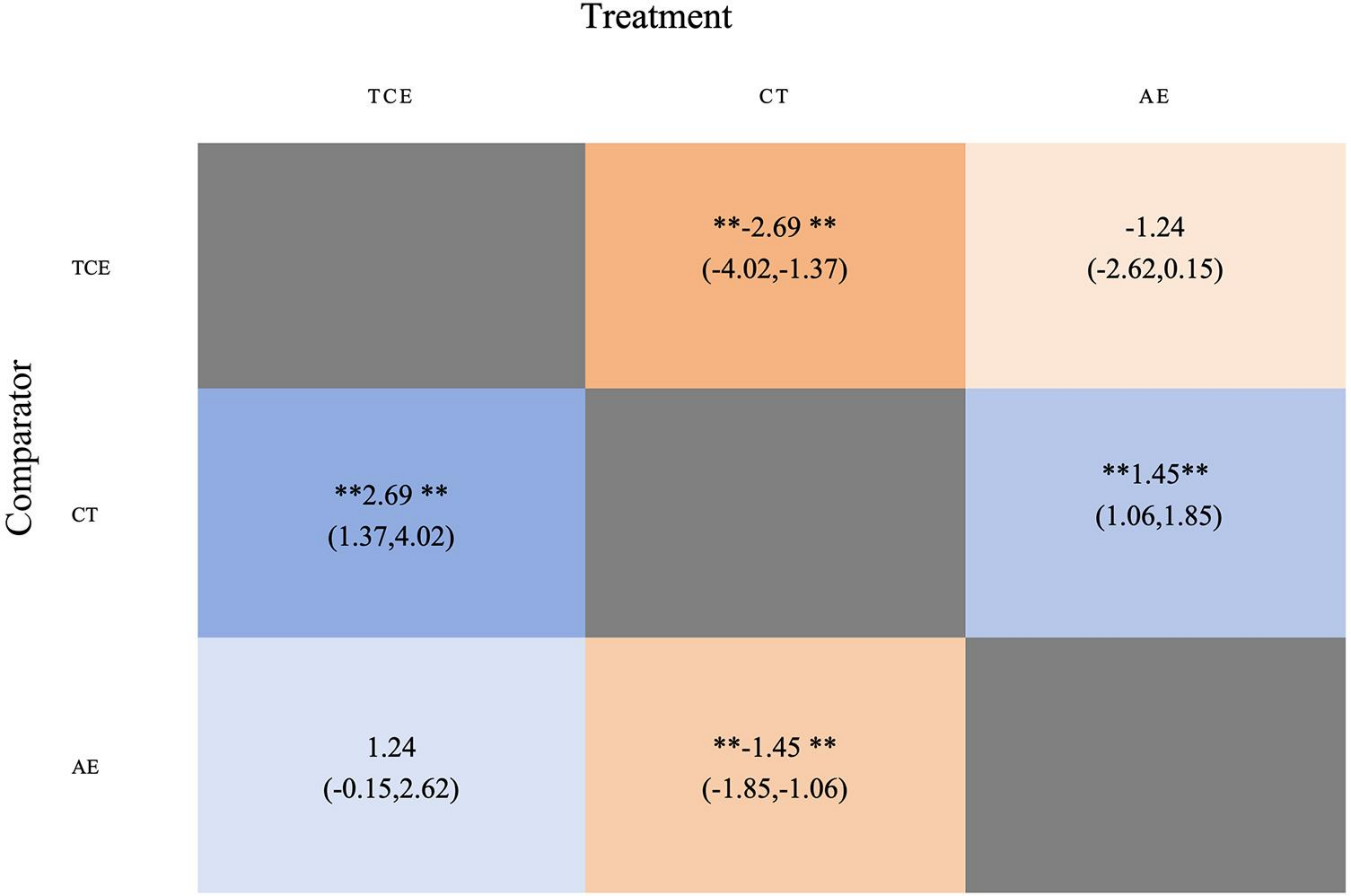
Network league plot of VO_2_peak (phase I).

The network meta-analysis revealed that compared to CT, both TCE [MD = 2.69, 95% CI = (1.37, 4.02)] and AE [MD = 1.45, 95% CI = (1.06, 1.85)] showed greater improvements than CT in improving VO_2_peak in patients with Stage I CHD. The probability ranking of different exercise interventions for improving VO_2_peak showed TCE ranked first in SUCRA (SUCRA: 98.0%, Table 1). Detailed pairwise comparisons between interventions are presented in Figure 4.

**Table 1:** Intervention ranking for VO_2_peak (phase I).

Intervention	VO_2_peak (Phase I)	
	SUCRA	Rank
TCE	98.0	1
AE	52.0	2
CT	0.0	3

##### 3.4.1.2 6MWD

**Figure 5:**
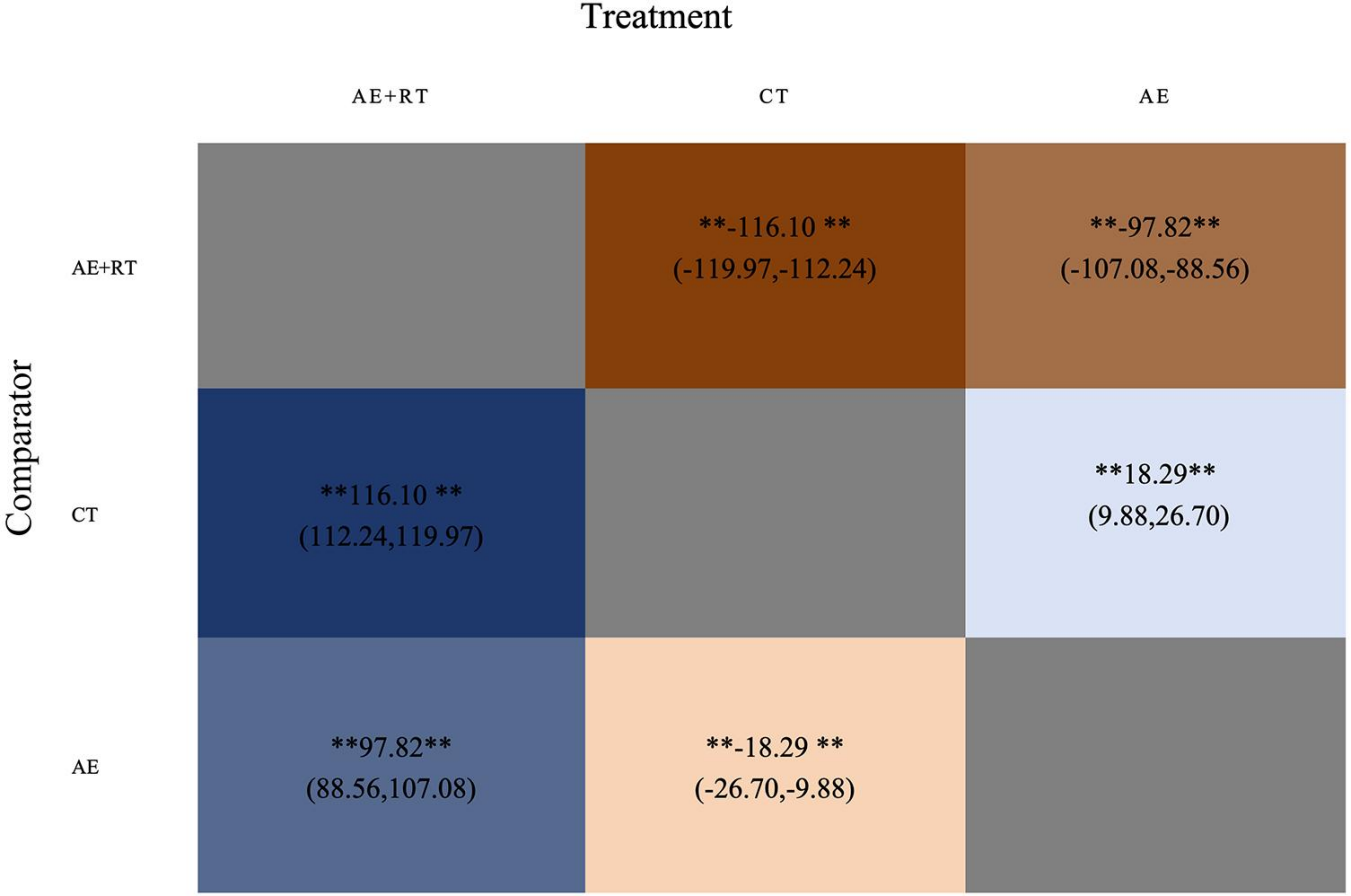
Network league plot of 6MWD (phase I).

The network meta-analysis results showed that compared with CT, both AE + RT [MD = 116.10, 95% CI = (112.24, 119.97)] and AE [MD = 18.29, 95% CI = (9.88, 26.70)] showed greater improvements than CT in improving 6MWD in patients with Stage I CHD. In addition, AE + RT was significantly superior to AE [MD = 97.82, 95% CI = (88.56, 107.08)]. The probability ranking of different exercise interventions for improving 6MWD showed AE ranked first in SUCRA (SUCRA: 75.7%, Table 2), followed by AE + RT (SUCRA: 60.7%), while CT had the lowest probability (SUCRA: 13.6%). Detailed pairwise comparisons between interventions are presented in Figure 5.

**Table 2:** Intervention ranking for 6MWD (phase I).

Intervention	6MWD (Phase I)	
	SUCRA	Rank
AE	75.7	1
AE + RT	60.7	2
CT	13.6	3

#### 3.4.2 Effects of exercise on CRF in phase II CHD

This study evaluated both direct and indirect evidence for each indicator in Stage II. The global inconsistency test showed *P* > 0*.*05 for VO_2_peak, indicating no statistical inconsistency; thus, the consistency model was used for pooling. The global inconsistency test for 6MWD yielded *P* *<* 0.05, indicating statistical inconsistency; therefore, the inconsistency model was employed for the pooled analysis. The details are shown in Supplementary Table S6.

##### 3.4.2.1 VO_2_peak

**Figure 6:**
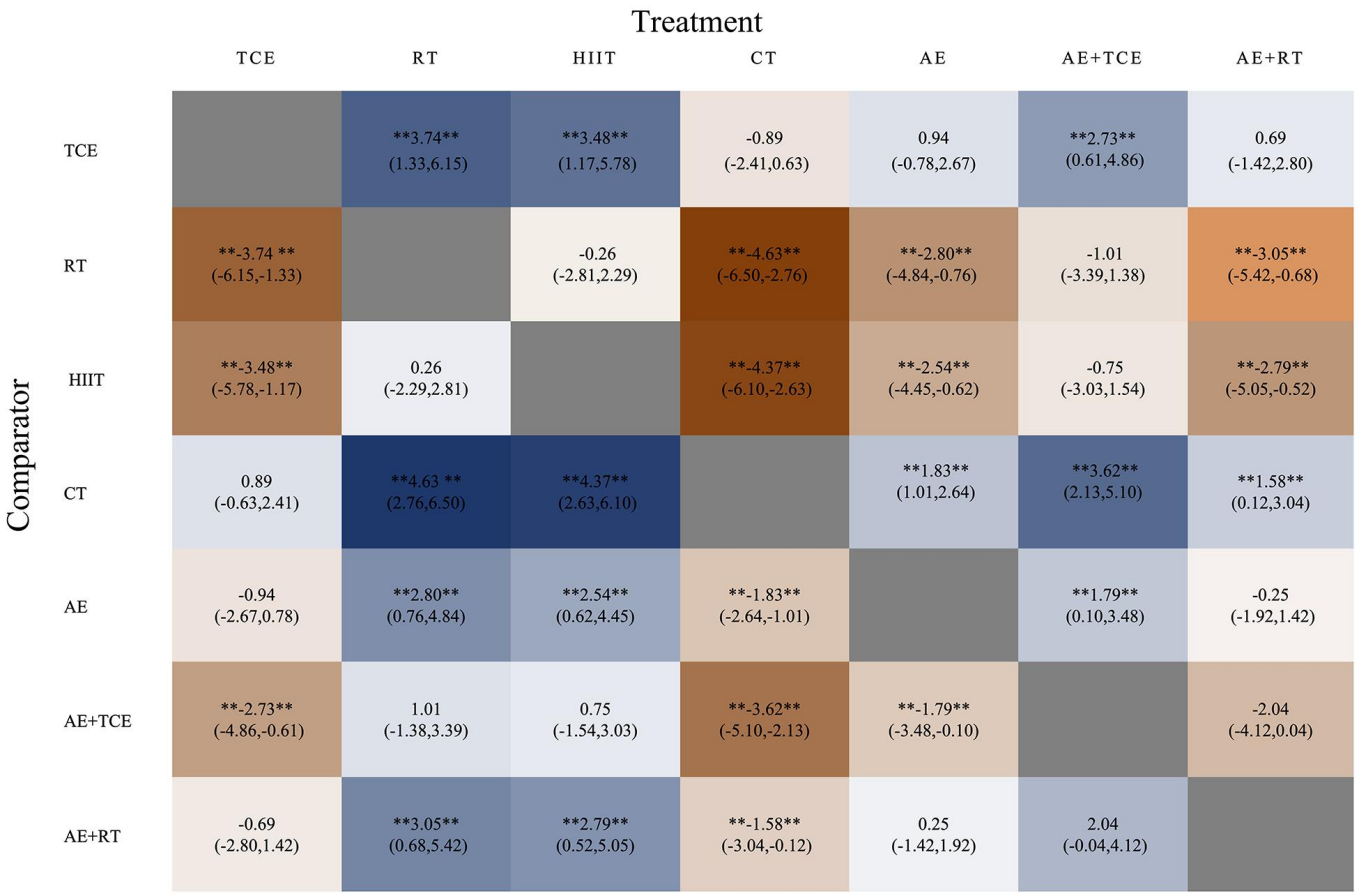
Network league plot of VO_2_peak (phase II).

The network meta-analysis results showed that compared with CT, RT [MD = 4.63, 95% CI = (2.76, 6.50)], HIIT [MD = 4.37, 95% CI = (2.63, 6.10)], AE + RT [MD = 1.58, 95% CI = (0.12, 3.04)], AE [MD = 1.83, 95% CI = (1.01, 2.64)], and AE + TCE [MD = 3.62, 95% CI = (2.13, 5.10)] significantly increased VO_2_peak in patients with stage II CHD; however, the difference between TCE and CT was not significant [MD = 0.89, 95% CI = (−0.63, 2.41)]. The probability ranking of different exercise interventions for improving VO_2_peak showed RT ranked first in SUCRA (SUCRA: 89.6%, Table 3), followed by HIIT (SUCRA: 85.7%), with AE + TCE in third place (SUCRA: 73.2%). The order of other interventions is shown in Table 3. Detailed pairwise comparison results are presented in Figure 6, and the direct comparison forest plot is shown in Supplementary Figure S1.

**Table 3:** Intervention ranking of VO_2_peak (phase II).

Intervention	VO_2_peak (Phase II)	
	SUCRA	Rank
RT	89.6	1
HIIT	85.7	2
AE + TCE	73.2	3
AE	41.5	4
AE + RT	36.2	5
TCE	21.2	6
CT	2.4	7

##### 3.4.2.2 6MWD

**Figure 7:**
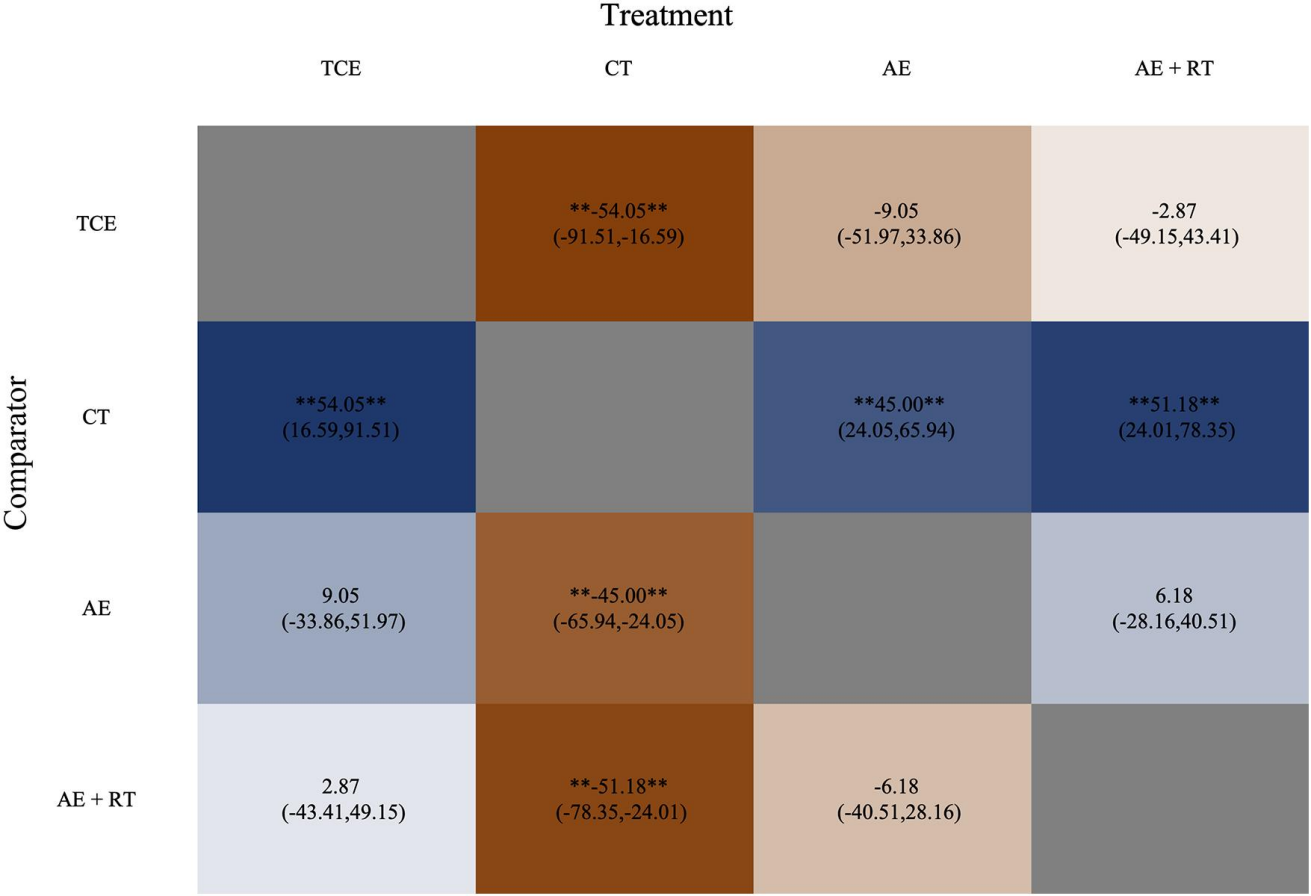
Network league plot of the 6MWD (phase II).

The results of the network meta-analysis showed that, compared with CT, TCE [MD = 54.05 m, 95% CI = (16.59, 91.51)], AE [MD = 45.00 m, 95% CI = (24.05, 65.94)], and AE + RT [MD = 51.18 m, 95% CI = (24.01, 78.35)] improved 6MWD in stage II patients. The point estimates for all three interventions exceeded the prespecified MCID of 25 m, suggesting potentially clinically meaningful improvements in addition to statistical significance. However, because the network showed statistical inconsistency and some confidence intervals were close to the MCID threshold, the magnitude of these effects should be interpreted cautiously. SUCRA ranking results indicated that TCE had the highest probability of benefit (SUCRA: 73.1%, Rank 1), followed closely by AE and AE + RT (69.7 and 57.0, Rank 2–3, respectively), while CT had the lowest probability (0.1, Rank 4). Detailed rankings are presented in Table 4. Due to limited available studies on AE + TCE for this outcome, a stable network ranking could not be established. The direct effect sizes and 95% CIs for each intervention compared with CT are visually presented in the forest plot (Supplementary Figure S1), while pairwise comparisons between interventions are detailed in Figure 7.

**Table 4:** Intervention ranking for 6MWD (phase II).

Intervention	6MWD (Phase II)	
	SUCRA	Rank
TCE	73.1	1
AE	69.7	2
AE + RT	57.0	3
CT	0.1	4
AE + TCE	/	/

#### 3.4.3 Effects of exercise on CRF in phase III CHD

This study evaluated both direct and indirect evidence for each indicator in Stage III. Global inconsistency testing revealed *P* < 0.05 for VO_2_peak, indicating statistical inconsistency, thus requiring consolidation using an inconsistent model; *P* > 0.05 for 6MWD showed no statistical inconsistency, thus allowing consolidation using a consistent model. The details are shown in Supplementary Table S6.

##### 3.4.3.1 VO_2_peak

**Figure 8:**
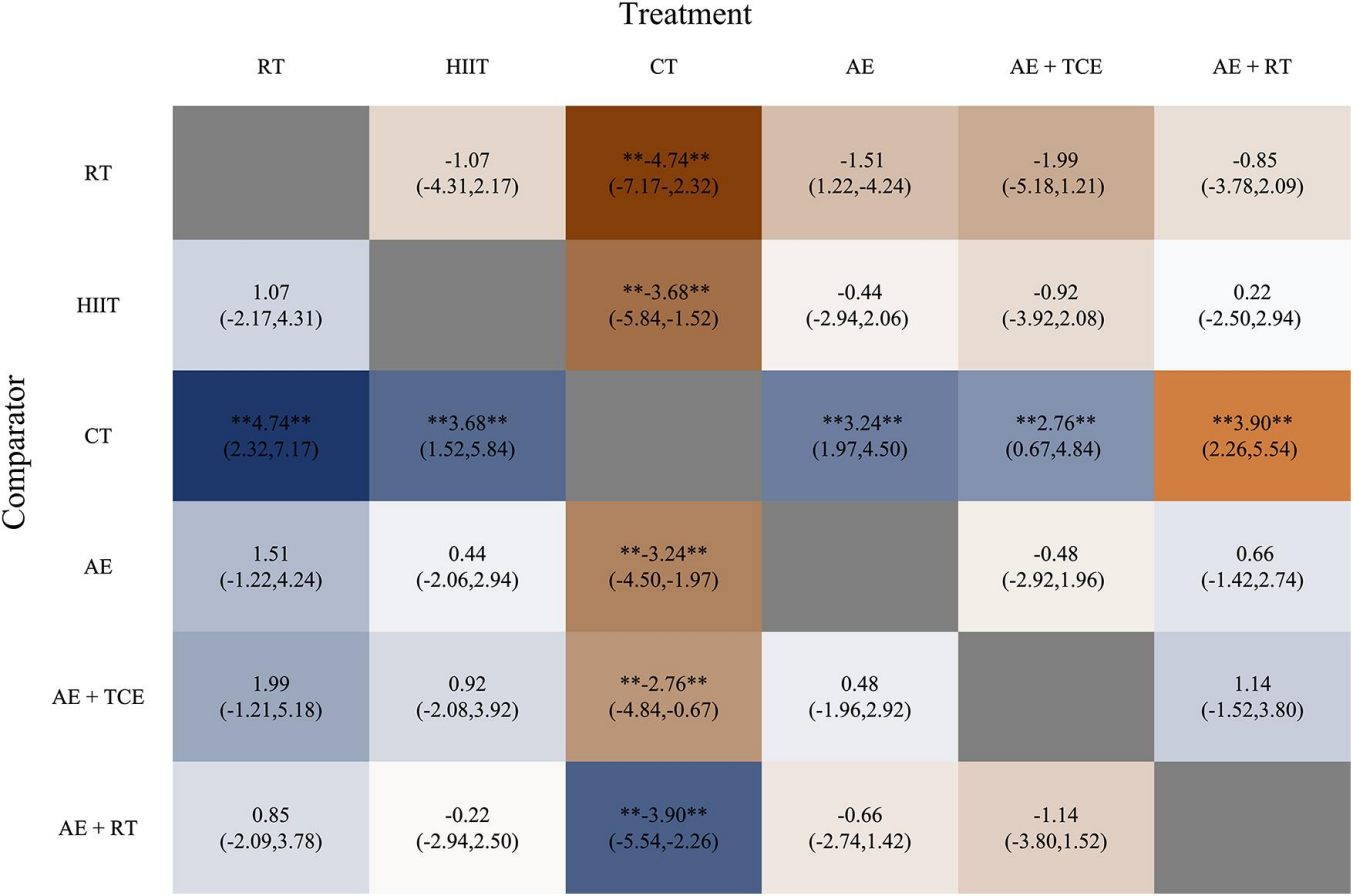
Network league plot of VO_2_peak (phase III).

The results of the network meta-analysis indicate that compared to CT, RT [MD = 4.74, 95% CI = (2.32, 7.17)], AE + RT [MD = 3.90, 95% CI = (2.26, 5.54)], HIIT [MD = 3.68, 95% CI = (1.52, 5.84)], AE [MD = 3.24, 95% CI = (1.97, 4.50)], and AE + TCE [MD = 2.76, 95% CI = (0.67, 4.84)] significantly increased VO_2_peak in stage III patients. SUCRA ranking results showed: RT (SUCRA 83.9%, Rank 1) > AE + RT (67.7%, Rank 2) > HIIT (61.7%, Rank 3) > AE (48.5%, Rank 4) > AE + TCE (38.1%, Rank 5) > CT (0.1%, Rank 6); Detailed ranking results are presented in Table 5, pairwise comparisons are shown in Figure 8, and direct comparison forest plots are available in Supplementary Figure S2.

**Table 5:** Intervention ranking of VO_2_peak (phase III).

Intervention	VO_2_peak (Phase III)	
	SUCRA	Rank
RT	83.9	1
AE + RT	67.7	2
HIIT	61.7	3
AE	48.5	4
AE + TCE	38.1	5
CT	0.1	6
TCE	/	/

##### 3.4.3.2 6MWD

**Figure 9:**
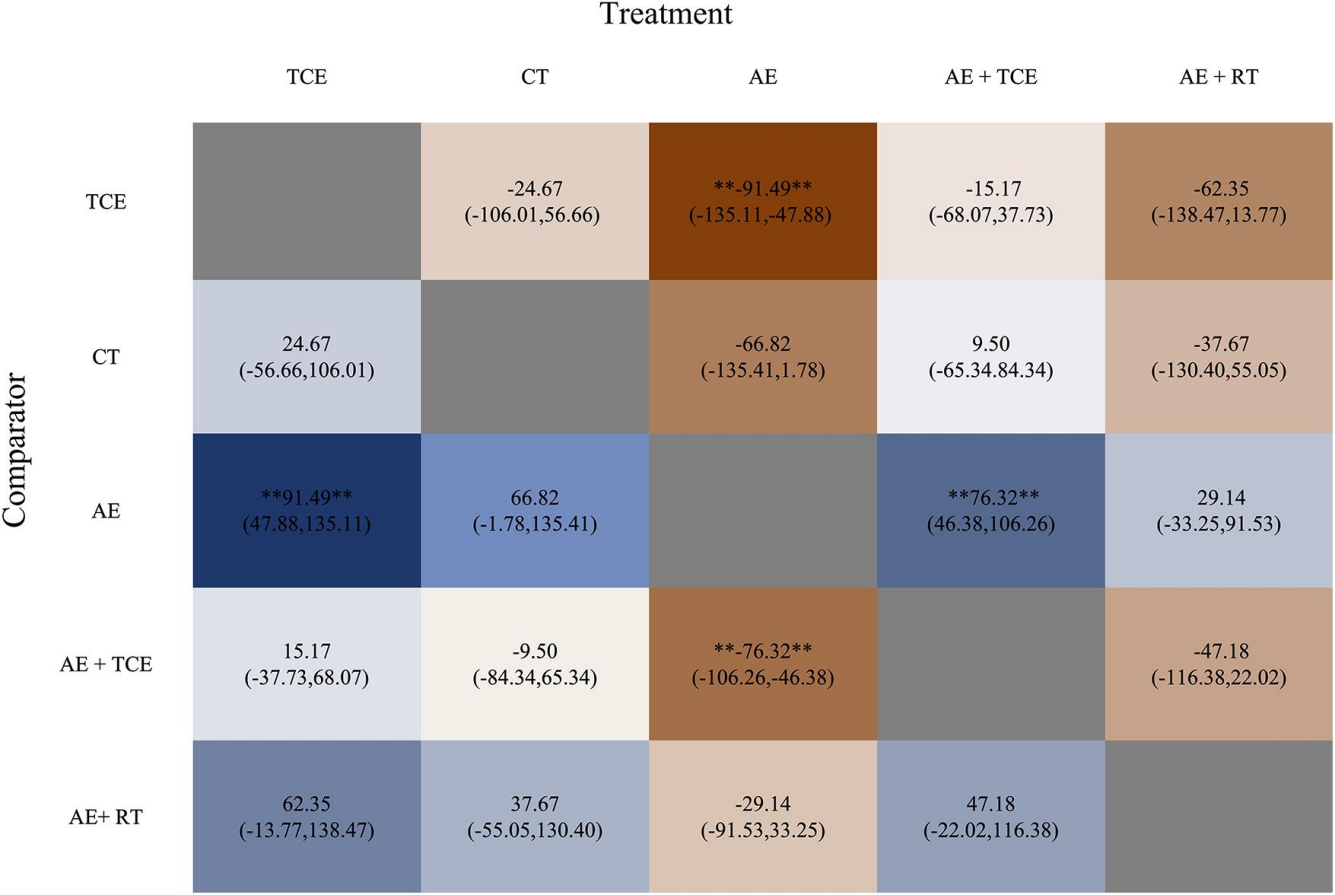
Network league plot of 6MWD (phase III).

The results of the network meta-analysis showed that, compared with CT, TCE [MD = 66.82 m, 95% CI = (1.78, 135.41)], AE [MD = 76.32 m, 95% CI = (46.38, 106.26)], AE + TCE [MD = 29.14 m, 95% CI = (−33.25, 91.53)], and AE + RT [MD = 91.49 m, 95% CI = (47.88, 135.11)] improved 6MWD in stage III patients, although most comparisons had wide confidence intervals. When interpreted against the prespecified MCID of 25 m, AE and AE + RT showed statistically significant and clinically meaningful improvements because both their point estimates and lower confidence limits exceeded the MCID threshold. TCE also had a point estimate above the MCID, but the lower confidence limit was below 25 m, indicating uncertainty in the magnitude of clinical benefit. AE + TCE should be interpreted cautiously because its point estimate only slightly exceeded the MCID and its 95% CI crossed zero. SUCRA ranking indicated that AE + RT had the highest probability of benefit for the phase III 6MWD outcome (SUCRA: 84.5%, Rank 1), followed by AE and TCE in second and third places (SUCRA: 70.1% and 61.1%), respectively. AE + TCE ranked fourth (SUCRA: 29.1%). Detailed rankings are shown in Table 6. Pairwise comparisons are detailed in Figure 9, with direct comparison forest plots shown in Supplementary Figure S2.

**Table 6:** Intervention ranking of the 6MWD (phase III).

Intervention	6MWD (Phase III)	
	SUCRA	Rank
AE + RT	84.5	1
AE	70.1	2
TCE	61.1	3
AE + TCE	29.1	4
CT	5.2	5

### 3.5 Publication bias assessment

Funnel plots were constructed for outcome measures across different stages of coronary heart disease to assess publication bias. Results showed generally symmetrical distribution of study points, with no significant evidence of publication bias. The network meta-analysis demonstrated robust results. The details are shown in Supplementary Figure S6.

### 3.6 Certainty of evidence

The GRADE approach assessment indicated that the quality of evidence for VO_2_peak and 6MWD was predominantly low to moderate. For the overall results, the primary factors contributing to downgrading the evidence grade were risk of bias within studies, imprecision, and small-sample effects. Specifically, risk of bias within studies significantly influenced the network estimates due to the inclusion of some high-risk studies; while imprecision was evident in the wide 95% confidence intervals for certain comparisons (e.g., AE + RT vs. CT improving 6MWD), indicating insufficient data to fully determine the precise magnitude of clinical effects. Furthermore, substantial small-sample effects were observed across multiple outcome measures, further limiting the certainty of evidence. Detailed assessment processes for evidence quality grading and specific scores for each domain are provided in Supplementary Table S7.

## 4 Discussion

This study focused on the effects of different exercise intervention modalities on cardiopulmonary endurance improvement in CHD patients across distinct cardiac rehabilitation phases. We included 87 RCTs involving 7,241 participants, covering six distinct exercise interventions (AE, RT, AE + RT, HIIT, TCE, AE + TCE) with conventional care (CT) as the control group. Through this comprehensive network meta-analysis, we found that the relative ranking of exercise modalities varied by rehabilitation phase and outcome measure. Overall, RT showed the highest ranking probability for improving VO_2_peak in phases II and III, while TCE ranked highest in phase I. Regarding 6MWD, AE ranked highest in phase I, TCE showed the highest ranking probability in phase II, and AE + RT appeared to be the most promising approach in phase III. These phase-specific patterns may reflect changes in clinical stability, hemodynamic tolerance, functional reserve, and exercise goals across rehabilitation, from protected early mobilization in Phase I to structured progressive training in Phase II and long-term combined training in Phase III (2).

We reviewed studies evaluating cardiopulmonary function and exercise tolerance in coronary artery disease patients, confirming that improved cardiopulmonary fitness correlates closely with better prognosis. In this context, VO_2_peak and 6MWD are the most commonly used and clinically significant indicators for assessing cardiac rehabilitation outcomes (118, 119). VO_2_peak is considered the gold standard for assessing cardiopulmonary fitness and a strong predictor of cardiovascular mortality (118), while 6MWD serves as a practical indicator of functional exercise capacity, better reflecting patients’ ability to perform activities of daily living (119). Together with evidence supporting the validity, reliability, and responsiveness of the 6MWT, this supports the use of 6MWD as a clinically meaningful functional outcome in the present analysis (22, 23). Therefore, the combined interpretation of VO_2_peak and 6MWD provides a more comprehensive assessment of both physiological adaptation and patient-relevant functional improvement after cardiac rehabilitation.

Regarding the key indicator VO_2_peak, our results indicate that in Phase I, TCE (SUCRA 98%) showed the highest ranking probability for improving VO_2_peak, followed by AE (52%), although this finding should be interpreted cautiously because of the limited evidence base and significant inconsistency. In Phase I, patients may still have hemodynamic vulnerability, post-procedural limitations, or reduced exercise tolerance; therefore, low-intensity, rhythmical, and easily supervised activities may be more feasible than demanding training regimens. During Phases II and III, RT showed the highest SUCRA ranking probability compared with other interventions. In Phase II, RT ranked first in SUCRA (89.6%), followed by HIIT (85.7%); in Phase III, RT remained first (83.9%), followed by AE + RT and HIIT. These findings may reflect the increasing importance of peripheral muscular adaptation as patients progress from early recovery to structured outpatient and maintenance rehabilitation. In Phase II, structured aerobic training with progressively introduced resistance training may improve VO_2_peak through both central cardiovascular adaptations and peripheral skeletal-muscle oxygen utilization, consistent with FITT-VP-based exercise progression (120). Although VO_2_peak is typically regarded as an indicator of aerobic capacity, RT may contribute to oxygen uptake by increasing muscle mass, improving muscular endurance, and enhancing peripheral oxygen extraction and utilization (19, 121). Concurrently, HIIT's high ranking across both phases supports its potential value, likely through central cardiovascular adaptations such as increased stroke volume and improved vascular endothelial function driven by high-intensity stimuli (122).

Beyond physiological changes in VO_2_peak, another core goal of cardiac rehabilitation is improving patients' functional status, typically measured by 6MWD. We observed a shift in the highest-ranked intervention across rehabilitation phases. In Phase I, the network suggested that AE had the highest ranking (SUCRA 75.7%), followed by AE + RT; however, this finding should be interpreted cautiously because of the limited number of studies and significant inconsistency. Early mobilization and low-intensity aerobic activity remain clinically important in Phase I because patients may still have hemodynamic vulnerability and limited exercise tolerance. In Phase II, TCE showed the highest ranking probability for improving 6MWD (SUCRA 73.1%, ranked first). This may relate to TCE (e.g., Tai Chi, Baduanjin) emphasizing mind-body integration, breath control, balance coordination, and low-to-moderate intensity movement, which may be more acceptable and sustainable for recently discharged patients (123, 124). In Phase III, as patients regain fitness and transition to long-term maintenance, AE + RT appeared to be the most promising approach (SUCRA 84.5%, ranked first). The combination of aerobic and resistance components may help maintain both cardiovascular fitness and musculoskeletal function, thereby supporting long-term walking capacity. When interpreted against the prespecified MCID of 25 m, the 6MWD improvements for AE + RT in Phase I, TCE, AE, and AE + RT in Phase II, and AE and AE + RT in Phase III may be clinically meaningful, with AE + RT showing particularly large point estimates in Phase I (116.10 m) and Phase III (91.49 m). Nevertheless, estimates with wide confidence intervals or confidence intervals crossing zero should be interpreted cautiously.

In the maintenance phase, technology-assisted or hybrid CR may further support long-term adherence through remote monitoring, goal setting, individualized feedback, and professional or peer support (125). Evidence from home-based cardiac rehabilitation suggests broadly similar benefits to centre-based programmes for exercise capacity and health-related quality of life (13). Wearable sensor-assisted home-based programmes may also indicate potential benefits for CRF and may complement conventional centre-based CR (14). In this context, structured telerehabilitation protocols provide examples of how wearable sensors, real-time supervision, and objective monitoring can be incorporated to guide exercise prescription, adherence, and safety (15). Although our network nodes were based on exercise modality rather than delivery setting, this body of evidence suggests that the practical use of AE, TCE, RT, HIIT, and AE + RT may vary across centre-based, home-based, and telerehabilitation contexts. This issue may be particularly important for higher-intensity or resistance-based modalities, which often require closer progression, technique monitoring, and safety supervision.

## 5 Strengths and limitations

First, our study possesses significant strengths. It incorporated 87 studies and over 7,200 participants, constructing an evidence network covering multiple mainstream and traditional exercise prescriptions, with standard care as the primary reference point for comparison. More importantly, by structuring independent evidence networks along cardiac rehabilitation phases, we provided more targeted and clinically relevant evidence-based recommendations than a general pooled analysis.

Second, this study has several limitations. The GRADE assessment indicates that the current evidence quality for VO_2_peak and 6MWD is predominantly low to moderate. The primary downgrading factors stem from the risk of bias within the original studies, imprecision in some comparison estimates (wide confidence intervals), and small-sample effects observed across multiple outcome measures. These factors limit the certainty of the results to some extent. Additionally, although we constructed independent evidence networks for different stages to reduce heterogeneity, statistical inconsistencies were detected in specific network comparisons (e.g., stage II 6MWD vs. stage III VO_2_peak). Potential sources of inconsistency may include variations in baseline patient characteristics, phase definitions, exercise intensity and duration, supervision level, country, clinical setting, and usual-care protocols across studies. Therefore, caution is warranted when interpreting these particular findings. A further limitation is the geographical concentration of the evidence base: 66 of the 87 included trials originated from China. This imbalance may affect generalizability to Western or non-Chinese cardiac rehabilitation settings. Traditional Chinese exercise modalities, such as Tai Chi, Qigong, and Baduanjin, may be more familiar and culturally acceptable in Chinese populations, potentially improving adherence and influencing the ranking of TCE-related interventions. Moreover, rehabilitation protocols, supervision models, referral pathways, usual-care practices, healthcare systems, and patient baseline characteristics may differ between China and Western countries. Therefore, these findings should be applied cautiously to non-Chinese clinical contexts, and further trials from diverse healthcare systems are needed.

Finally, readers should interpret our ranking results with caution because the evidence networks were largely star-shaped. Most comparisons were indirectly connected through CT, with few direct head-to-head comparisons between active exercise modalities. This structure limits the ability to examine local inconsistency through node-splitting and may reduce the certainty of SUCRA-based rankings. In addition, although interventions were categorized by exercise modality, differences in exercise prescription dose were not fully explored. Important FITT-VP components, including frequency, intensity, session duration, total training volume, and progression strategies, may substantially influence physiological adaptation and may have contributed to heterogeneity across studies. Future research should therefore compare not only exercise modality but also exercise dose and progression, preferably through high-quality, large-sample head-to-head RCTs, to clarify the optimal prescription strategy across cardiac rehabilitation phases.

## 6 Conclusion

This network meta-analysis suggests that structured exercise interventions generally provide greater improvements in cardiopulmonary endurance than standard care across different rehabilitation phases for patients with coronary heart disease. However, the relative ranking of exercise modalities appears to depend on both the rehabilitation phase and the outcome measure. Based on current evidence, TCE showed the highest ranking probability for improving VO_2_peak in Phase I and enhancing 6MWD in Phase II; RT ranked highest for improving VO_2_peak during Phases II and III; and AE + RT appeared to be the most promising approach for improving 6MWD in Phase III. These results support phase-tailored, individualized exercise prescriptions in cardiac rehabilitation and suggest that a one-size-fits-all strategy may be inappropriate. However, given the low-to-moderate certainty of evidence, geographical imbalance of the included trials, limited direct head-to-head comparisons, and imprecision in some estimates, clinicians should interpret these rankings cautiously and tailor exercise prescriptions to individual patient characteristics, safety considerations, delivery settings, and available supervision. Further high-quality studies from diverse healthcare systems are needed to validate these findings.

## Data Availability

The raw data supporting the conclusions of this article will be made available by the authors, without undue reservation.
