# Supplementary material for: Optimal exercise prescription for patients with coronary heart disease across cardiac rehabilitation phases: a systematic review and network meta-analysis
Source: Front Sports Act Living. 2026 Jun 11;8:1813426. doi: 10.3389/fspor.2026.1813426 (PMC13294071; doi:10.3389/fspor.2026.1813426)
Supplement: Supplementary file 1 [file Datasheet1.pdf]

Supplementary Material

1 Supplementary Data

2 Supplementary Figures and Tables

Supplementary Figures

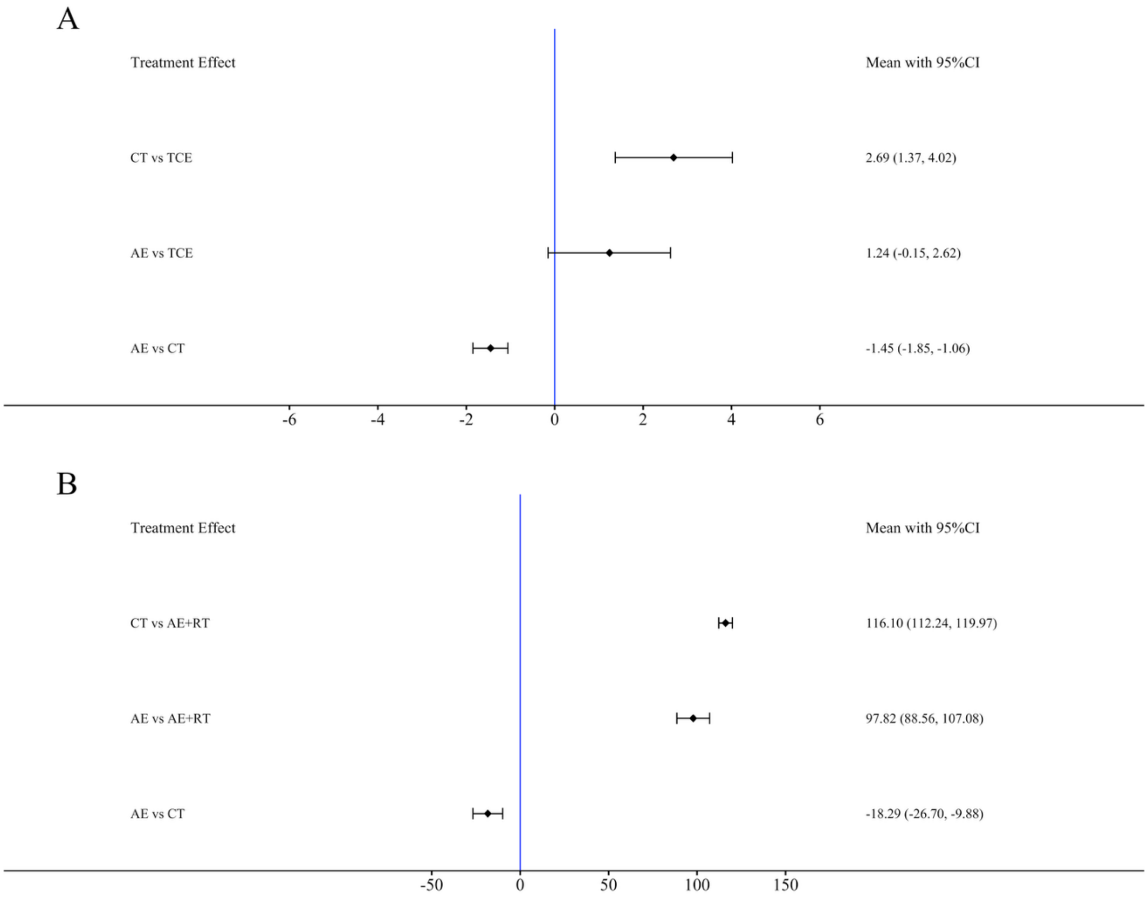

Supplementary Figure 1. Forest plot of Phase I: (A) VO_2peak, (B) 6MWD. AE, aerobic exercise; AE+RT, aerobic exercise + resistance training; TCE, traditional Chinese exercise; CT, conventional therapy.

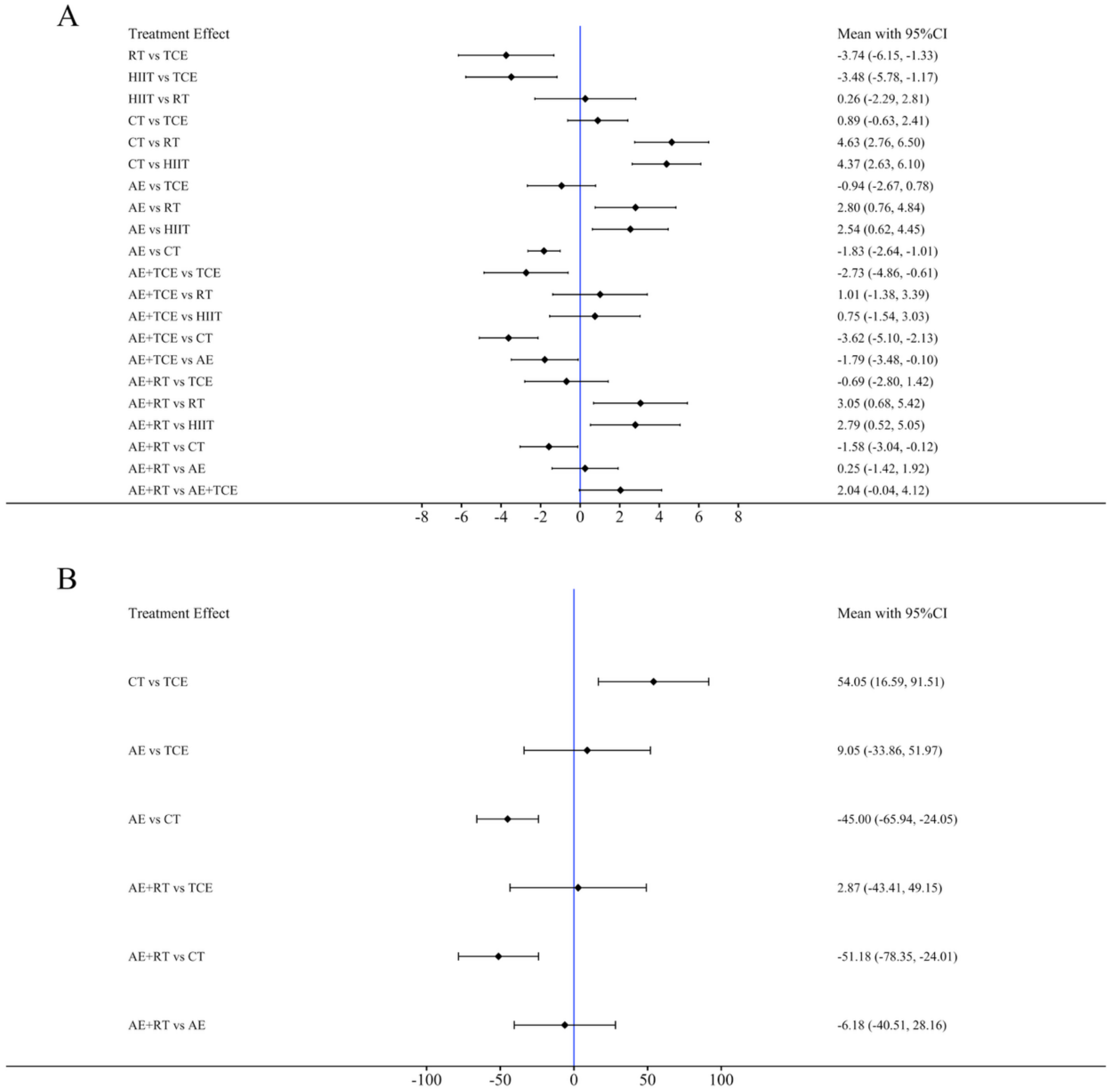

Supplementary Figure 2. Forest plot of Phase II: (A) VO_2peak, (B) 6MWD. AE, aerobic exercise; RT, resistance training; AE+RT, aerobic exercise + resistance training; HIIT, high-intensity interval

training; TCE, traditional Chinese exercise; AE+TCE, aerobic exercise + traditional Chinese exercise; CT, conventional therapy.

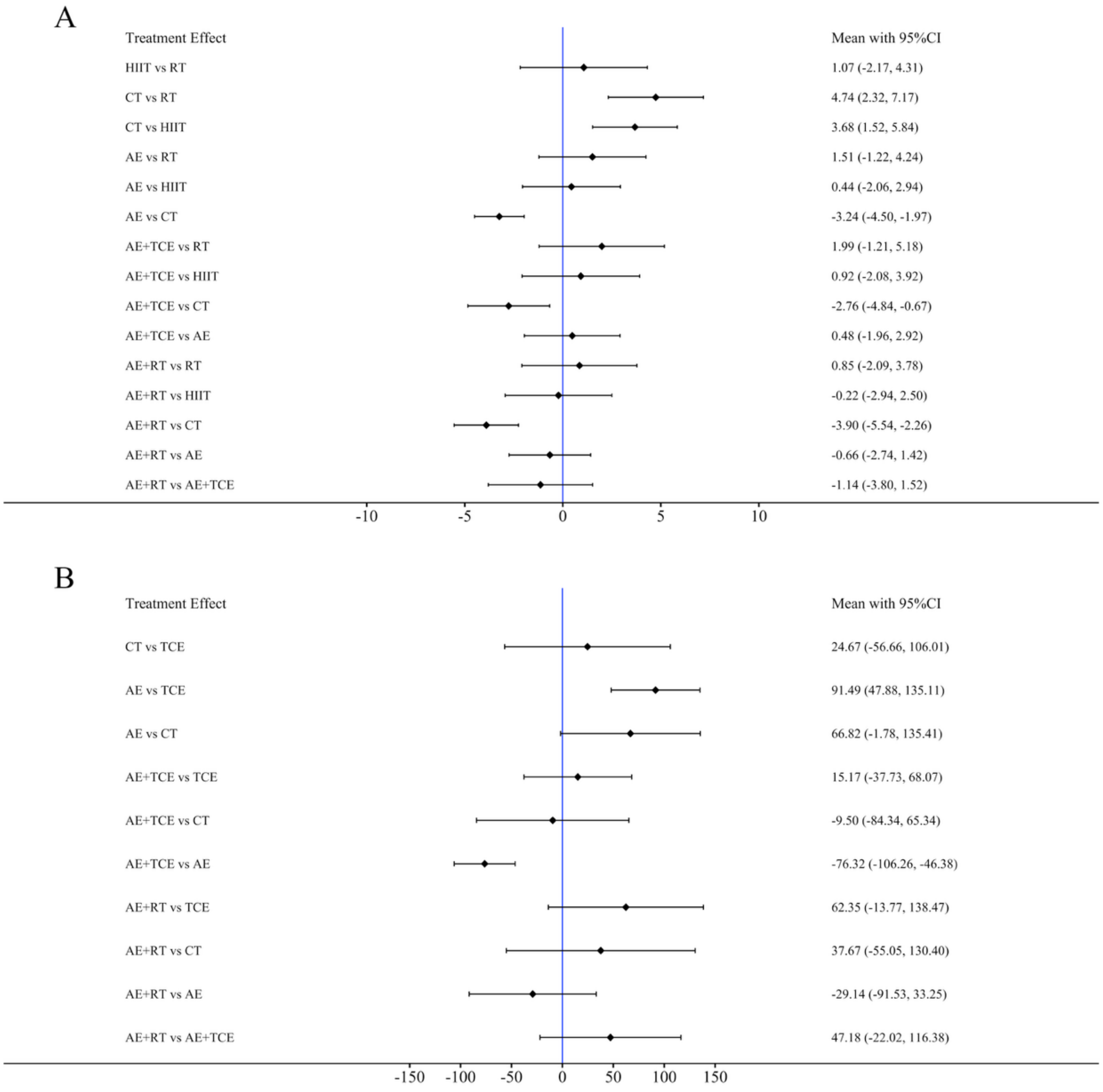

Supplementary Figure 3. Forest plot of Phase III: (A) VO_2peak, (B) 6MWD. AE, aerobic exercise; RT, resistance training; AE+RT, aerobic exercise + resistance training; HIIT, high-intensity interval training; TCE, traditional Chinese exercise; AE+TCE, aerobic exercise + traditional Chinese exercise; CT, conventional therapy.

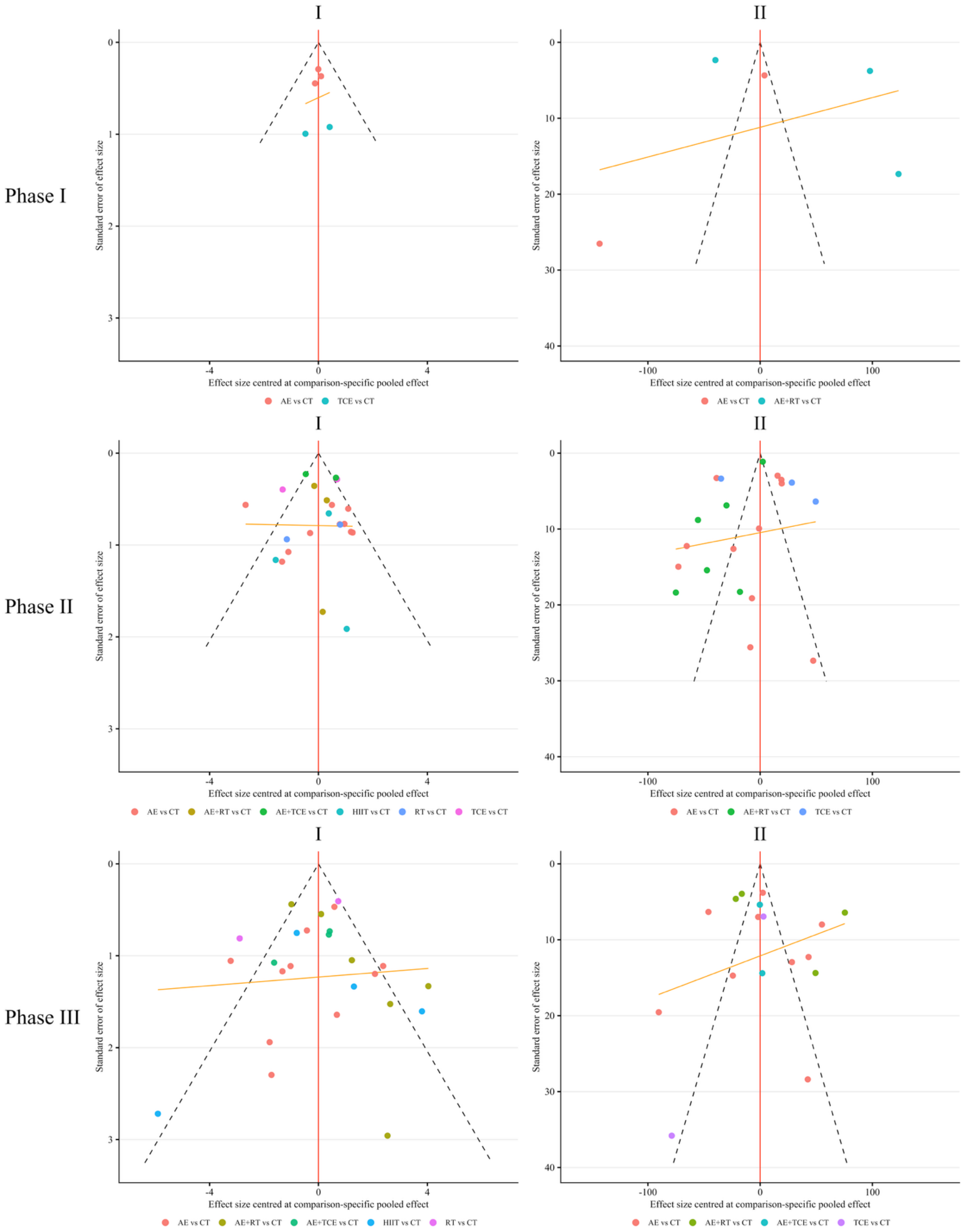
I
II
Phase I
Standard error of effect size
Effect size centred at comparison-specific pooled effect
AE vs CT
TCE vs CT
AE vs CT
AE+RT vs CT
Phase II
AE vs CT
AE+RT vs CT
AE+TCE vs CT
HIIT vs CT
RT vs CT
TCE vs CT
AE vs CT
AE+RT vs CT
TCE vs CT
Phase III
AE vs CT
AE+RT vs CT
AE+TCE vs CT
HIIT vs CT
RT vs CT
AE vs CT
AE+RT vs CT
AE+TCE vs CT
TCE vs CT

Supplementary Figure 4. Funnel plots: (I) VO_2peak, (II) 6MWD.

Supplementary Tables

Supplementary Table 1. Search strategies of Chinese databases (CNKI and Wanfang Database)

Fields: subject/topic. Filters and limits followed each database interface (humans; trials where applicable).

Search ID	Search terms / logical combination
#1.	"冠心病"[主题]
#2.	"冠状动脉粥样硬化性心脏病"[主题]
#3.	"缺血性心脏病"[主题]
#4.	"CAD"[主题]
#5.	"CHD"[主题]
#6.	OR/1-5
#7.	"有氧运动"[主题]
#8.	"有氧训练"[主题]
#9.	"抗阻训练"[主题]
#10.	"力量训练"[主题]
#11.	"高强度间歇训练"[主题]
#12.	"HIIT"[主题]
#13.	"中国传统功法"[主题]
#14.	"太极"[主题]
#15.	"八段锦"[主题]
#16.	"气功"[主题]
#17.	"五禽戏"[主题]
#18.	"导引养生功"[主题]
#19.	"有氧+抗阻"[主题]
#20.	"有氧结合抗阻"[主题]
#21.	"AE+RT"[主题]
#22.	"有氧+传统功法"[主题]
#23.	"aerobic"[主题]
#24.	"resistance training"[主题]
#25.	"tai chi"[主题]
#26.	"qigong"[主题]
#27.	"baduanjin"[主题]
#28.	OR/7-27
#29.	"峰值摄氧量"[主题]
#30.	"VO2peak"[主题]
#31.	"VO2max"[主题]
#32.	"最大摄氧量"[主题]
#33.	"6 分钟步行距离"[主题]
#34.	"6MWD"[主题]
#35.	"6MWT"[主题]
#36.	"6 分钟步行试验"[主题]
#37.	OR/29-36
#38.	"随机对照试验"[主题]
#39.	"随机"[主题]
#40.	"对照"[主题]

#41.	"试验"[主题]
#42.	"randomized"[主题]
#43.	"controlled trial"[主题]
#44.	"clinical trial"[主题]
#45.	OR/38-44
#46.	#6 AND #28 AND #37 AND #45

Supplementary Table 2. Search strategies of PubMed (adapted for Embase, Cochrane Library, Web of Science, Scopus, and EBSCO)

Fields: MeSH and Title/Abstract. Filters: humans; clinical trials/randomized terms; languages Chinese/English; up to April 30 2026.

Search ID	Search terms / logical combination
#1.	"Coronary Artery Disease"[Mesh]
#2.	"Myocardial Ischemia"[Mesh]
#3.	"coronary artery disease"[tiab]
#4.	"ischemic heart disease"[tiab]
#5.	CAD[tiab]
#6.	OR/1-5
#7.	"Exercise Therapy"[Mesh]
#8.	"Exercise"[Mesh]
#9.	aerobic[tiab]
#10.	"aerobic training"[tiab]
#11.	"resistance training"[tiab]
#12.	"strength training"[tiab]
#13.	HIIT[tiab]
#14.	"high-intensity interval training"[tiab]
#15.	"traditional Chinese exercise*"[tiab]
#16.	"tai chi"[tiab]
#17.	taiji[tiab]
#18.	qigong[tiab]
#19.	baduanjin[tiab]
#20.	"aerobic plus resistance"[tiab]
#21.	"AE+RT"[tiab]
#22.	"AE plus RT"[tiab]
#23.	OR/7-22
#24.	"Oxygen Consumption"[Mesh]
#25.	"Exercise Test"[Mesh]
#26.	"VO2peak"[tiab]
#27.	"VO2 max"[tiab]
#28.	"peak oxygen"[tiab]
#29.	"6-minute walk"[tiab]
#30.	"six minute walk"[tiab]
#31.	6MWD[tiab]
#32.	6MWT[tiab]
#33.	OR/24-32
#34.	randomized controlled trial[pt]
#35.	controlled clinical trial[pt]
#36.	randomized[tiab]
#37.	randomised[tiab]

#38.	"clinical trial"[tiab]
#39.	OR/34-38
#40.	NOT (animals[mh] NOT humans[mh])
#41.	#6 AND #23 AND #33 AND #39
#42.	#41 AND #40

Supplementary Table 3. Search strategies of MEDLINE (via Ovid)

Fields: exp subject headings and ti,ab. Limits: humans; English or Chinese; Search updated to April 30, 2026.

Search ID	Search terms / logical combination
1.	exp Coronary Artery Disease/
2.	exp Myocardial Ischemia/
3.	(coronary artery disease or ischemic heart disease or CAD).ti,ab.
4.	or/1-3
5.	exp Exercise Therapy/
6.	exp Exercise/
7.	(aerobic or "aerobic training" or "resistance training" or "strength training" or HIIT or "high-intensity interval training" or "tai chi" or qigong or baduanjin or "aerobic plus resistance" or "AE+RT").ti,ab.
8.	or/5-7
9.	exp Oxygen Consumption/
10.	exp Exercise Test/
11.	exp Walk Test/
12.	(VO2peak or "VO2 max" or "oxygen consumption" or "6-minute walk" or 6MWD or 6MWT).ti,ab.
13.	or/9-12
14.	randomized controlled trial.pt.
15.	controlled clinical trial.pt.
16.	(randomized or randomised or trial).ti,ab.
17.	or/14-16
18.	4 and 8 and 13 and 17
19.	limit 18 to humans
20.	limit 19 to (English or Chinese)
21.	Search updated to April 30, 2026

Supplementary Table 4. Definitions of exercise interventions

Intervention type	Abbreviation	Definition used in this review
Aerobic exercise	AE	Rhythmic, large-muscle dynamic exercise (e.g., walking, cycling, treadmill, cycle ergometer) primarily prescribed to improve cardiorespiratory fitness. Intensity was generally prescribed at light-to-vigorous levels (approximately 40–80% of heart-rate reserve or VO_2peak), for about 20–60 minutes per session, on at least 3 days per week. Aerobic programmes using continuous or interval formats that did not meet the predefined HIIT criteria were classified as AE. When aerobic training included only warm-up/cool-down, stretching, balance, calisthenics, or very light strengthening activities without a structured resistance-training

Intervention type	Abbreviation	Definition used in this review
		prescription, the node was retained as AE rather than AE+RT.
Resistance training	RT	Structured strength-training targeting major muscle groups using weight machines, free weights, elastic bands, body weight, or comparable resistance devices. Typical prescription involved 1–3 sets of 8–15 repetitions at approximately 30–80% of one-repetition maximum, or a clearly described progressive resistance load, on at least 2 non-consecutive days per week. Programmes were classified as RT only when the exercise intervention was dominated by resistance training without a planned aerobic component.
Aerobic exercise + Resistance training	AE + RT	Multicomponent programmes that integrated both an aerobic component and a structured resistance-training component within the same session or training week. The aerobic component followed the AE definition, and the resistance component had to include a planned strength-training prescription with defined exercises and at least one of the following: intensity/load, sets, repetitions, progression, or training frequency. Programmes with aerobic exercise plus only mild, incidental, or non-progressive strengthening were not classified as AE+RT unless the original study explicitly defined these activities as a resistance-training component.
High-intensity interval training	HIIT	Intermittent aerobic training consisting of repeated bouts of high-intensity exercise interspersed with active or passive recovery. HIIT was distinguished from general AE when the original prescription clearly described interval-based training with high-intensity bouts, typically ≥80–90% of peak heart rate, heart-rate reserve, VO_2peak, peak workload, or a comparable symptom-limited peak intensity. Moderate-intensity continuous training, moderate interval training, or interval aerobic training not meeting these high-intensity criteria was classified as AE.
Traditional Chinese exercises	TCE	Low- to moderate-intensity mind–body exercise modalities originating from traditional Chinese practices, characterised by slow and continuous whole-body movements, coordinated diaphragmatic breathing, and focused attention (e.g., Tai Chi, Qigong, Baduanjin). Programmes were classified as TCE when the intervention consisted mainly of TCE as the stand-alone structured exercise component, either alone or in addition to usual care. Simple walking or conventional aerobic warm-up performed only as preparation for TCE was not considered sufficient to form an AE+TCE node.
Aerobic exercise + Traditional Chinese exercises	AE + TCE	Exercise programmes that combined conventional aerobic exercise (e.g., walking, cycling, treadmill, or cycle ergometer training) with TCE within the same rehabilitation plan, either in the same session or on alternating days. AE+TCE was distinguished from TCE alone only when the conventional aerobic component was clearly planned and prescribed as part of the intervention, with identifiable frequency, duration, intensity, or progression. Programmes centred on TCE with only brief warm-up, cool-down, or

Intervention type	Abbreviation	Definition used in this review
		unspecific walking were classified as TCE rather than AE+TCE.

Operational rule for unclear prescriptions: Two reviewers independently classified each intervention node according to the above FITT-based rules. If the original exercise prescription was unclear, the classification was first determined from the reported exercise type, intensity, session content, progression, and stated intervention aim. Disagreements were resolved through discussion; when consensus could not be reached, a third reviewer was consulted. If the available information remained insufficient after discussion, the intervention was assigned to the most conservative compatible node based on its dominant exercise component.

Supplementary Table 5. Characteristics of the Included Studies

Study	Country	Participants (IG/CG)	Age (years)	Phase	Intervention comparison	Exercise dose	Outcome
Aktan ÖO 2026 [79]	Turkey	16/13	IG: 52.5 ± 5.4<br>CG: 55.9 ± 8.0	II	AE + RT vs CT	40 min/session, 3 sessions/week, 8 weeks	6MWD
Avila A 2018 [49]	Belgium	30/26	IG: 58.6 ± 13<br>CG: 61.9 ± 7.3	II	AE + RT vs CT	50 min/session, 3 sessions/week, 12 weeks	VO_2peak
Bai M 2022 [53]	China	63/63	IG: 65.4 ± 6.8<br>CG: 66.9 ± 7.2	II	AE vs CT	20–40 min/session, 3 sessions/week, 12 weeks	VO_2peak
Belardinelli R 2001 [97]	USA	59/59	IG: 52 ± 11<br>CG: 59 ± 10	III	AE vs CT	50 min/session, 3 sessions/week, 24 weeks	VO_2peak
Bilińska M 2010 [59]	Poland	60/60	IG: 54.1 ± 5.8<br>CG: 53.9 ± 5.0	II	AE vs CT	60 min/session, 3 sessions/week, 6 weeks	VO_2peak
Cai C 2022 [94]	China	49/48	IG: 57 ± 11<br>CG: 57 ± 9	III	AE vs CT	65 min/session, 5 sessions/week, 12 weeks	VO_2peak
Cai Y 2022 [38]	China	45/45	IG: 49.58 ± 9.41<br>CG: 49.47 ± 9.32	I	TCE vs CT	50 min/session, 5–7 sessions/week, 6 weeks	6MWD, VO_2peak
Cao RY 2021 [55]	China	24/10	IG: 67.2 ± 1.9<br>CG: 59.4 ± 2.4	II	AE vs CT	30 min/session, 3 sessions/week, 8 weeks	VO_2peak
Cepicka L 2026 [40]	Czechia	25/25	IG: 53.48 ± 10.58<br>CG: 58.52 ± 7.8	I	AE vs CT	20 min/session, 10 sessions/week, 4 weeks	6MWD

Study	Country	Participants (IG/CG)	Age (years)	Phase	Intervention comparison	Exercise dose	Outcome
Chen BH 2019 [104]	China	46/46	IG: 60.03 ± 11.08<br>CG: 59.88 ± 10.82	III	AE vs CT	30–45 min/session, 3–5 sessions/week, 12 weeks	6MWD
Chen CH 2014 [73]	Taiwan, China	21/15	IG: 69.7 ± 4.5<br>CG: 69.0 ± 4.6	II	AE + RT vs CT	50–60 min/session, 3 sessions/week, 12 weeks	6MWD
Chen F 2022 [86]	China	30/30	IG: 63.74 ± 8.21<br>CG: 61.87 ± 8.54	III	AE + TCE vs CT	30–60 min/session, 3 sessions/week, 12 weeks	VO_2peak
Chen H 2023 [80]	China	33/33	IG: 62.3 ± 8.3<br>CG: 67.8 ± 7.2	III	HIIT vs CT	40–60 min/session, 3 sessions/week, 12 weeks	VO_2peak
Chen PJ 2018 [69]	China	30/30	IG: 57.20 ± 6.40<br>CG: 55.40 ± 7.10	II	AE vs CT	20–45 min/session, 3–5 sessions/week, 24 weeks	6MWD
Clark IN 2017 [109]	Australia	27/26	IG: 67.3 ± 4.3<br>CG: 69.7 ± 8.3	III	AE vs CT	50 min/session, 3 sessions/week, 26 weeks	6MWD
Cui WJ 2021 [51]	China	43/43	IG: 54.83 ± 6.57<br>CG: 56.01 ± 7.82	II	HIIT vs CT	40 min/session, 3 sessions/week, 12 weeks	VO_2peak
Dong X 2021 [37]	China	47/47	IG: 65.74 ± 7.72<br>CG: 65.49 ± 7.55	I	AE vs CT	30 min/session, 4 sessions/week, 8 weeks	VO_2peak
Eder B 2010 [17]	Austria	41/19	IG: 73.1 ± 5.1<br>CG: 73.2 ± 3.7	II	AE vs CT	40 min/session, 3 sessions/week, 4 weeks	6MWD, VO_2peak
Fan QJ 2019 [18]	China	30/30	IG: 61.8 ± 8.1<br>CG: 60.8 ± 7.6	II	AE + RT vs CT	30–50 min/session, 3–5 sessions/week, 4 weeks	VO_2peak
Gao Y 2023 [19]	China	200/200	IG: 71.60 ± 9.32<br>CG: 72.00 ± 6.73	III	AE + RT vs CT	20–60 min/session, 3–4 sessions/week, 12 weeks	6MWD
Gao ZZ 2015 [20]	China	22/22	IG: 59.4 ± 7.9<br>CG: 60.4 ± 8.6	II	HIIT vs CT	40 min/session, 3 sessions/week, 12 weeks	VO_2peak

Study	Country	Participants (IG/CG)	Age (years)	Phase	Intervention comparison	Exercise dose	Outcome
Geng JY 2022 [21]	China	38/38	IG: 62.12 ± 10.12<br>CG: 63.01 ± 6.23	II	AE vs CT	30–60 min/session, 3 sessions/week, 12 weeks	6MWD, VO_2peak
Giallauria F 2012 [22]	Italy	24/26	IG: 54 ± 7<br>CG: 52 ± 10	II	AE vs CT	30 min/session, 3 sessions/week, 24 weeks	VO_2peak
Goncalves C 2024 [23]	Portugal	23/23	IG: 50 ± 9<br>CG:57 ± 11	III	HIIT vs CT	95 min/session, 3 sessions/week, 6 weeks	VO_2peak
Gu X 2021 [24]	China	55/55	IG: 67.15 ± 6.52<br>CG: 68.22 ± 6.47	III	AE + RT vs CT	20 min/session, 5 sessions/week, 12 weeks	VO_2peak
Hong XJ 2020 [25]	China	33/34	IG: 60.70 ± 7.82<br>CG: 60.24 ± 7.26	II	TCE vs CT	40 min/session, 5 sessions/week, 8 weeks	6MWD, VO_2peak
Januszek R 2023 [26]	Poland	13/15	IG: 60 ± 7.31<br>CG: 64 ± 6.07	II	AE vs CT	60 min/session, 2 sessions/week, 24 weeks	6MWD, VO_2peak
Kim HJ 2013 [27]	Korea	17/15	IG: 56.06 ± 7.31<br>CG: 54.33 ± 8.53	III	AE vs CT	50 min/session, 5 sessions/week, 6 weeks	VO_2peak
Lennon O 2008 [28]	Ireland	24/24	IG: 59.0 ± 10.3<br>CG: 60.5 ± 10.0	II	AE vs CT	30 min/session, 2 sessions/week, 10 weeks	VO_2peak
Li X 2023 [29]	China	54/54	IG: 55.69 ± 5.11<br>CG: 54.36 ± 5.85	III	AE vs CT	40–60 min/session, 3 sessions/week, 12 weeks	VO_2peak
Li Y 2022 [30]	China	40/40	IG: 70.56 ± 1.72<br>CG: 69.85 ± 2.07	III	AE + TCE vs CT	30 min/session, 5 sessions/week, 16 weeks	6MWD
Li YY 2021 [31]	China	49/48	IG: 68.15 ± 4.71<br>CG: 67.42 ± 4.26	III	AE vs CT	20–30 min/session, 3 sessions/week, 12 weeks	6MWD
Li Z 2023 [32]	China	40/40	IG: 55.4 ± 8.9<br>CG: 55.6 ± 8.3	II	AE + RT vs CT	20–30 min/session, 5–7 sessions/week, 24 weeks	6MWD
Li ZC 2019 [33]	China	40/40	IG: 55.32 ± 9.52	II	AE + RT vs CT	30–50 min/session, 3–5 sessions/week, 12 weeks	6MWD

Study	Country	Participants (IG/CG)	Age (years)	Phase	Intervention comparison	Exercise dose	Outcome
			CG: 56.47 ± 11.83				
Lin XF 2021 [34]	China	50/50	IG: 60.82 ± 14.23<br>CG: 60.86 ± 14.20	II	AE vs CT	30 min/session, 7 sessions/week, 12 weeks	6MWD
Lin XL 2023 [35]	China	40/40	IG: 57.07 ± 7.44<br>CG: 57.13 ± 7.51	I	AE + RT vs CT	30 min/session, 4 sessions/week, 4 weeks	6MWD
Liu HJ 2017 [36]	China	33/33	IG: 55.21 ± 1.32<br>CG: 54.76 ± 1.31	II	TCE vs CT	30 min/session, 3 sessions/week, 12 weeks	6MWD
Liu SQ 2023 [37]	China	50/50	IG: 66.09 ± 3.30<br>CG: 66.11 ± 3.28	II	AE + RT vs CT	60 min/session, 3–5 sessions/week, 12 weeks	VO_2peak
Liu SX 2017 [38]	China	35/38	IG: 58.4 ± 6.1<br>CG: 59.1 ± 6.9	III	AE + RT vs CT	60 min/session, 3 sessions/week, 12 weeks	VO_2peak
Liu SZ 2020 [39]	China	50/50	IG: 87.9 ± 5.1<br>CG: 88.6 ± 4.8	III	AE vs CT	15–30 min/session, 4 sessions/week, 12 weeks	6MWD
Liu XH 2018 [40]	China	35/35	IG: 61.13 ± 8.73<br>CG: 59.84 ± 7.98	III	RT vs CT	40 min/session, 2sessions/week, 12 weeks	VO_2peak
Long T 2021 [41]	China	30/30	IG: 51.6 ± 8.9<br>CG: 53.2 ± 9.2	II	AE + TCE vs CT	60 min/session, 3 sessions/week, 12 weeks	VO_2peak
Lu B 2022 [42]	China	33/35	IG: 52.27 ± 8.42<br>CG: 52.91 ± 8.89	III	AE + RT vs CT	20–40 min/session, 3 sessions/week, 12 weeks	6MWD, VO_2peak
Ma CJ 2020 [43]	China	15/15	IG: 61.40 ± 11.72<br>CG: 67.27 ± 8.35	III	TCE vs CT	60 min/session, 3 sessions/week, 12 weeks	6MWD
Madssen E 2014 [44]	Norway	24/25	IG: 64.4 ± 7.8<br>CG: 58.5 ± 7.3	III	HIIT vs CT	40–60 min/session, 3 sessions/week, 12 weeks	VO_2peak

Study	Country	Participants (IG/CG)	Age (years)	Phase	Intervention comparison	Exercise dose	Outcome
Mou J 2022 [45]	China	45/45	IG: 59.11 ± 4.98<br>CG: 59.02 ± 5.16	III	AE + RT vs CT	60 min/session, 4 sessions/week, 24 weeks	VO_2peak
Nam H 2024 [46]	Korea	29/32	IG: 58.69 ± 12.38<br>CG: 56.66 ± 9.50	II	HIIT vs CT	30 min/session, 2 sessions/week, 36 weeks	6MWD, VO_2peak
Ni XS 2021 [47]	China	60/60	IG: 55.20 ± 15.61<br>CG: 56.51 ± 18.15	III	TCE vs CT	30 min/session, 7 sessions/week, 24 weeks	6MWD
Ouyang YY 2017 [48]	China	20/20	IG: 54.3 ± 10.8<br>CG: 51.2 ± 11.4	III	AE + RT vs CT	50 min/session, 3 sessions/week, 48 weeks	6MWD, VO_2peak
Pedersen LR 2015 [49]	Denmark	26/29	IG: 62.3 ± 5.7<br>CG: 63.6 ± 6.8	III	AE vs CT	60 min/session, 3 sessions/week, 12 weeks	VO_2peak
Peydro ED 2022 [50]	Spain	31/28	IG: 57.5 ± 9.0<br>CG: 54.7 ± 9.9	III	AE + RT vs CT	40–60 min/session, 4 sessions/week, 40 weeks	VO_2peak
Qin Y 2021 [51]	China	52/52	IG: 65.84 ± 3.58<br>CG: 65.76 ± 3.52	I	AE + RT vs CT	30 min/session, 3 sessions/week, 4 weeks	6MWD
Rui HM 2021 [52]	China	55/55	IG: 55.2 ± 7.1<br>CG: 55.4 ± 7.6	III	AE + RT vs CT	30 min/session, 12 sessions/week, 7 weeks	6MWD
Salvetti XM 2008 [53]	Brazil	19/20	IG: 53 ± 8<br>CG: 54 ± 9	III	AE vs CT	30 min/session, 3 sessions/week, 12 weeks	VO_2peak
Scheer A 2021 [54]	Australia	15/12	IG: 66 ± 8<br>CG: 67 ± 8	III	AE + RT vs CT	60 min/session, 3 sessions/week, 12 weeks	VO_2peak
Seki E 2008 [55]	Japan	18/16	IG: 69 ± 3<br>CG: 70 ± 4	III	AE vs CT	20–60 min/session, 3 sessions/week, 24 weeks	VO_2peak
Snoek JA 2021 [56]	Netherlands	61/61	IG: 60.0 ± 8.4<br>CG: 59.0 ± 10.7	III	AE vs CT	30 min/session, 5 sessions/week, 24 weeks	VO_2peak

Study	Country	Participants (IG/CG)	Age (years)	Phase	Intervention comparison	Exercise dose	Outcome
Sun XJ 2019 [57]	China	40/40	IG: 55.29 ± 4.08<br>CG: 56.28 ± 3.46	II	AE vs CT	30–40 min/session, 3 sessions/week, 12 weeks	6MWD
Sun XJ 2020 [58]	China	40/40	IG: 56.63 ± 9.29<br>CG: 55.90 ± 8.33	II	AE + TCE vs CT	68 min/session, 3 sessions/week, 12 weeks	VO_2peak
Tao L 2022 [59]	China	75/75	IG: 51.8 ± 8.1<br>CG: 53.4 ± 7.2	III	AE vs CT	20–45 min/session, 3–5 sessions/week, 24 weeks	6MWD
Tian CW 2016 [60]	China	30/30	IG: 61.4 ± 6.9<br>CG: 60.5 ± 6.5	III	AE + TCE vs CT	60 min/session, 3–5 sessions/week, 12 weeks	6MWD
Vasiliauskas D 2007 [61]	Lithuania	83/71	IG: 58.49 ± 4.3<br>CG: 59.59 ± 5.2	III	AE vs CT	15–30 min/session, 14 sessions/week, 16 weeks	6MWD, VO_2peak
Wang HJ 2020 [62]	China	63/64	IG: 62.76 ± 2.61<br>CG: 63.17 ± 2.54	I	AE vs CT	15 min/session, 7 sessions/week, 4 weeks	VO_2peak
Wang L 2015 [63]	China	38/37	IG: 61.4 ± 8.9<br>CG: 63.0 ± 9.1	II	AE vs CT	30–60 min/session, 3 sessions/week, 12 weeks	VO_2peak
Wang L 2022 [64]	China	64/64	IG: 58.3 ± 6.9<br>CG: 56.8 ± 7.1	III	AE vs CT	30–40 min/session, 3–5 sessions/week, 24 weeks	6MWD
Wang X 2023 [65]	China	40/40	IG: 52.0 ± 16.7<br>CG: 52.5 ± 15.5	II	TCE vs CT	60 min/session, 7 sessions/week, 8 weeks	6MWD
Wang XL 2021 [66]	China	29/29	IG: 64.28 ± 5.64<br>CG: 64.38 ± 6.59	I	TCE vs CT	60 min/session, 5–7 sessions/week, 4 weeks	VO_2peak
Wang Y 2022 [67]	China	30/30	IG: 57.13 ± 9.91<br>CG: 58.23 ± 7.04	II	AE vs CT	40 min/session, 4 sessions/week, 12 weeks	6MWD
Wu Q 2021 [68]	China	60/60	IG: 56.43 ± 7.59<br>CG: 55.72 ± 6.85	II	AE vs CT	20–60 min/session, 3–5 sessions/week, 24 weeks	6MWD

Study	Country	Participants (IG/CG)	Age (years)	Phase	Intervention comparison	Exercise dose	Outcome
Xia YQ 2017 [69]	China	24/25	IG: 55.2 ± 7.1<br>CG: 53.7 ± 6.8	I	AE + RT vs CT	60 min/session, 3 sessions/week, 8 weeks	6MWD
Xian YL 2020 [70]	China	60/60	IG: 67.7 ± 5.8<br>CG: 67.5 ± 5.9	II	AE + RT vs CT	60 min/session, 3 sessions/week, 24 weeks	6MWD
Xu YM 2017 [71]	China	58/60	IG: 56.40 ± 8.12<br>CG: 58.62 ± 7.96	II	AE vs CT	30–40 min/session, 3 sessions/week, 24 weeks	6MWD
Xu YY 2023 [72]	China	98/98	IG: 60.54 ± 8.59<br>CG: 61.23 ± 8.74	II	AE + RT vs CT	30–60 min/session, 3–5 sessions/week, 24 weeks	6MWD
Yang L 2020 [73]	China	52/52	IG: 84.63 ± 3.58<br>CG: 85.58 ± 3.61	III	AE vs CT	30 min/session, 4 sessions/week, 12 weeks	6MWD, VO_2peak
Yang XH 2024 [74]	China	24/24	IG: 66.17 ± 6.46<br>CG: 67.92 ± 7.30	II	AE vs CT	20 min/session, 5 sessions/week, 12 weeks	VO_2peak
Ye YH 2021 [75]	China	40/40	IG: 58.96 ± 9.30<br>CG: 58.94 ± 9.23	III	AE + TCE vs CT	15–20 min/session, 4–6 sessions/week, 12 weeks	VO_2peak
Yin ZZ 2020 [76]	China	50/50	IG: 68.64 ± 4.27<br>CG: 68.52 ± 4.13	III	AE vs CT	30 min/session, 3–5 sessions/week, 12 weeks	6MWD
You WJ 2017 [77]	China	42/42	IG: 64.3 ± 6.1<br>CG: 63.9 ± 6.2	II	RT vs CT	60 min/session, 3 sessions/week, 12 weeks	VO_2peak
Yu ML 2018 [78]	China	60/60	IG: 60.33 ± 8.78<br>CG: 60.68 ± 9.70	II	TCE vs CT	45 min/session, 2–3 sessions/week, 12 weeks	6MWD, VO_2peak
Zhai SD 2022 [79]	China	30/30	IG: 60.07 ± 6.82<br>CG: 57.73 ± 8.03	II	AE vs CT	30–45 min/session, 4 sessions/week, 12 weeks	6MWD
Zhang Y 2018 [80]	China	65/65	IG: 70.3 ± 10.7<br>CG: 69.8 ± 10.4	III	AE vs CT	60 min/session, 3–5 sessions/week, 12 weeks	6MWD
Zhao F 2018 [81]	China	30/29	IG: 61.23 ± 5.24	III	TCE vs CT	40–60 min/session, 6–7 sessions/week, 12 weeks	VO_2peak

Study	Country	Participants (IG/CG)	Age (years)	Phase	Intervention comparison	Exercise dose	Outcome
			CG: 60.66 ± 5.85				
Zhao M 2022 [82]	China	50/50	IG: 68.49 ± 6.17<br>CG: 69.85 ± 4.36	III	AE + RT vs CT	30–40 min/session, 4–5 sessions/week, 12 weeks	6MWD
Zhao Y 2016 [83]	China	32/32	IG: 59.3 ± 7.1<br>CG: 60.5 ± 9.7	III	HIIT vs CT	30 min/session, 3 sessions/week, 12 weeks	VO_2peak
Zheng XW 2019 [84]	China	46/46	IG: 59.82 ± 10.02<br>CG: 60.25 ± 10.21	II	RT vs CT	30–40 min/session, 3 sessions/week, 12 weeks	6MWD, VO_2peak
Zhou F 2016 [85]	China	29/26	IG: 61.4 ± 8.9<br>CG: 62.3 ± 8.7	II	AE vs CT	30–60 min/session, 5 sessions/week, 12 weeks	VO_2peak
Zhu YH 2024 [86]	China	50/50	IG: 57.96 ± 9.30<br>CG: 57.94 ± 9.23	I	AE vs CT	25–55 min/session, 3 sessions/week, 24 weeks	6MWD, VO_2peak

Abbreviations: IG, intervention group; CG, control group. AE, aerobic exercise; RT, resistance training; AE+RT, aerobic exercise + resistance training; HIIT, high-intensity interval training; TCE, traditional Chinese exercise; AE + TCE, aerobic exercise + traditional Chinese exercise; CT, conventional therapy.

Supplementary Table 6. Global inconsistency test results and model selection. 6MWD, 6-minute walk distance; VO_2peak, peak oxygen uptake; CT, conventional therapy.

Outcome	Rehabilitation phase	Global inconsistency test (P value)	Model used
VO_2peak	Phase I	0.0001	Inconsistency model
VO_2peak	Phase II	0.2522	Consistency model
VO_2peak	Phase III	0.0001	Inconsistency model
6MWD	Phase I	0.0001	Inconsistency model
6MWD	Phase II	0.0047	Inconsistency model
6MWD	Phase III	0.0562	Consistency model

Supplementary Table 7. GRADE evidence. Certainty symbols: High (⊕⊕⊕⊕), Moderate (⊕⊕⊕◯), Low (⊕⊕◯◯), Very low (⊕◯◯◯)

Outcome	Comparison (intervention vs CT)	k	N	Effect (MD, 95% CI)	Certainty	Reasons for downgrading
VO_2peak (Phase I; mL·kg⁻¹·min⁻¹)	TCE vs CT	2	138	2.69 (1.37 to 4.02)	Low (⊕⊕◯◯)	Imprecision; RoB; Small-study effects
VO_2peak (Phase I; mL·kg⁻¹·min⁻¹)	AE vs CT	3	321	1.45 (1.06 to 1.85)	Moderate (⊕⊕⊕◯)	RoB; Small-study effects
6MWD (Phase I; m)	AE vs CT	2	150	85.55 (-58.32 to 229.42)	Low (⊕⊕◯◯)	Imprecision; RoB; Small-study effects
6MWD (Phase I; m)	AE + RT vs CT	3	234	56.34 (-54.44 to 167.11)	Low (⊕⊕◯◯)	Imprecision; RoB; Small-study effects
VO_2peak (Phase II; mL·kg⁻¹·min⁻¹)	RT vs CT	2	176	4.63 (2.71 to 6.55)	Moderate (⊕⊕⊕◯)	RoB; Small-study effects
VO_2peak (Phase II; mL·kg⁻¹·min⁻¹)	HIIT vs CT	3	191	4.30 (2.99 to 5.62)	Moderate (⊕⊕⊕◯)	RoB; Small-study effects
VO_2peak (Phase II; mL·kg⁻¹·min⁻¹)	AE + TCE vs CT	2	140	3.62 (2.54 to 4.71)	Moderate (⊕⊕⊕◯)	RoB; Small-study effects
VO_2peak (Phase II; mL·kg⁻¹·min⁻¹)	AE vs CT	10	631	1.83 (0.88 to 2.78)	Moderate (⊕⊕⊕◯)	RoB; Small-study effects
VO_2peak (Phase II; mL·kg⁻¹·min⁻¹)	AE + RT vs CT	3	216	1.66 (1.09 to 2.22)	Moderate (⊕⊕⊕◯)	RoB; Small-study effects
VO_2peak (Phase II; mL·kg⁻¹·min⁻¹)	TCE vs CT	2	179	0.90 (-1.06 to 2.86)	Moderate (⊕⊕⊕◯)	RoB; Small-study effects
6MWD (Phase II; m)	TCE vs CT	3	254	53.90 (2.68 to 105.12)	Moderate (⊕⊕⊕◯)	RoB; Small-study effects
6MWD (Phase II; m)	AE vs CT	11	870	45.01 (25.63 to 64.38)	Moderate (⊕⊕⊕◯)	RoB; Small-study effects
6MWD (Phase II; m)	AE + RT vs CT	6	544	51.18 (24.01 to 78.35)	Moderate (⊕⊕⊕◯)	RoB; Small-study effects
VO_2peak (Phase III; mL·kg⁻¹·min⁻¹)	RT vs CT	2	180	4.82 (1.28 to 8.37)	Moderate (⊕⊕⊕◯)	RoB; Small-study effects
VO_2peak (Phase III; mL·kg⁻¹·min⁻¹)	AE + RT vs CT	6	360	3.96 (2.47 to 5.45)	Moderate (⊕⊕⊕◯)	RoB; Small-study effects
VO_2peak (Phase III; mL·kg⁻¹·min⁻¹)	HIIT vs CT	4	219	3.80 (0.96 to 6.63)	Moderate (⊕⊕⊕◯)	RoB; Small-study effects
VO_2peak (Phase III; mL·kg⁻¹·min⁻¹)	AE vs CT	10	794	3.21 (2.14 to 4.27)	Moderate (⊕⊕⊕◯)	RoB; Small-study effects
VO_2peak (Phase III; mL·kg⁻¹·min⁻¹)	AE + TCE vs CT	3	199	2.62 (1.49 to 3.75)	Moderate (⊕⊕⊕◯)	RoB; Small-study effects
6MWD (Phase III; m)	AE + RT vs CT	4	676	91.45 (46.36 to 136.53)	Moderate (⊕⊕⊕◯)	RoB; Small-study effects

Outcome	Comparison (intervention vs CT)	k	N	Effect (MD, 95% CI)	Certainty	Reasons for downgrading
6MWD (Phase III; m)	AE vs CT	9	954	76.25 (51.69 to 100.81)	Moderate (⊕⊕⊕◯)	RoB; Small-study effects
6MWD (Phase III; m)	TCE vs CT	2	90	69.05 (-9.66 to 147.76)	Low (⊕⊕◯◯)	Imprecision; RoB; Small-study effects
6MWD (Phase III; m)	AE + TCE vs CT	2	140	29.92 (20.04 to 39.80)	Moderate (⊕⊕⊕◯)	RoB; Small-study effects

Abbreviations: CT, conventional therapy; VO_2peak, peak oxygen uptake; 6MWD, 6-minute walk distance; AE, aerobic exercise; RT, resistance training; AE+RT, aerobic exercise plus resistance training; HIIT, high-intensity interval training; TCE, traditional Chinese exercise; AE+TCE, aerobic exercise plus traditional Chinese exercise; RoB, risk of bias. k indicates the number of studies contributing to each comparison, and N indicates the total number of participants. VO_2peak is reported in $mL \cdot kg^{-1} \cdot min^{-1}$, and 6MWD is reported in meters.
